# Supplementary material for: Genotype imputation accuracy and the quality metrics of the minor ancestry in multi-ancestry reference panels
Source: Brief Bioinform. 2024 Jan 13;25(1):bbad509. doi: 10.1093/bib/bbad509 (PMC10788679; doi:10.1093/bib/bbad509)
Supplement: revised_supp_v2_bbad509 [file revised_supp_v2_bbad509.docx]

**Supplementary Methods**

**Supplementary Method 1. Cohort specification of the Biobank Japan (BBJ), genotyping, QC, and selection of EAS subjects**

The BBJ project first cohort enrolled 200k participants from 2003 to 2007 [1,2]. Subjects were genotyped by either the Illumina HumanOmniExpressExome BeadChip or a combination of the Illumina HumanOmniExpress and HumanExome BeadChips. The coordinates were on the genome build hg19. QC steps were described in the previous literature [3]. Briefly, variants with (1) call rate < 99%; (2) p-value for Hardy Weinberg equilibrium (HWE) < 1e-6; (3) number of heterozygotes < 5 were excluded. Additional QC was performed by comparing the genotypes between the whole genome sequencing (WGS) and the array of 939 subjects, and then variants with a concordance rate < 99.5% or a non-reference discordance rate ≥ 0.5% were excluded, as described previously. After QC, 520,378 variants on autosomes remained. Samples with a call rate < 98% were excluded. The ancestries were determined by projecting the principal component (PC) onto the PC space of the 1KGP, and 180,882 individuals of the EAS ancestry were used in this study. We named it BBJ-180k.

**Supplementary Method 2. Methods to construct the BBJ1k and JEWEL3k reference panels**

Methods to construct the BBJ1k reference panel were described in the previous literature [3]. Briefly, 1,037 samples from the BBJ were sequenced at 30 × depth. After calling the variants using GATK (v3.2-2) and performing QC, variants located at the low-complexity regions (LCR; accessed from ftp://ftp.1000genomes.ebi.ac.uk/vol1/ftp/release/20130502/supporting/low_complexity_regions/hs37d5-LCRs.20140224.bed.gz) were removed. BEAGLE was used to impute the missing genotypes. To combine the WGS dataset with the 1KGP, variants located at the multi-allelic sites were removed. Then SHAPEIT was used for phasing, and IMPUTE2 was used to combine them. Singletons and variants at multi-allelic sites were removed from the final merged reference panel. Methods to construct the JEWEL3k reference panel were described in the previous literature [4]. Briefly, a total of 1,491 and 1,765 samples from BBJ were sequenced at 30 × and 15 × depth, respectively. The same process with the BBJ1k was used to construct the JEWEL3k reference panel. One sample in BBJ1k was excluded from the JEWEL3k due to the joint QC.

**Supplementary Method 3. QC, liftover, and variant comparison using the external WGS dataset**

The 1,007 samples with WGS were used to evaluate the imputation quality. Peripheral blood-derived genomic DNA was extracted, prepared by TruSeq Nano DNA Library Preparation Kit, and sequenced at 15 × depth using Illumina HiSeq 2500 (150bp paired-ends). Genotypes were called using BWA-MEM (v0.7.13) and GATK (v3.8). The reference genome was hg19 (build37+decoy from the 1KGP; ftp://ftp-trace.ncbi.nih.gov/1000genomes/ftp/technical/reference/phase2_reference_assembly_sequence/hs37d5.fa.gz). We applied the quality filter of GQ ≥ 20 and DP ≥ 2. Variants with a missing rate > 10% were removed. To avoid the bias caused by the cryptic relatives between the reference panel and target sample, PLINK v1.9 was used to calculate the PI_HAT (using variants on the array which were pruned by Plink with “--indep-pairwise 200 50 0.35” and with MAF ≥ 10%) in a combination of the JEWEL3k and 1,007 WGS samples. No pairs were above 0.1. Fourteen samples were excluded as not in all imputed datasets. We named it WGS_993_. Chromosome 19 was selected as the representative as no variants were on the known inverted regions (by comparing to the UCSC LiftOver chain file; https://hgdownload.soe.ucsc.edu/goldenPath/hg38/liftOver/) between hg38 and hg19. Only variants that could be lifted interchangeably (not to the alternative contig or duplicated position) between hg19 and hg38 were retained. There were 581,172 SNVs and 65,157 indels remaining. For SNVs, we compared the alternative allele frequency (AAF) between the TOPMed imputation result and WGS_993_, then swapped the ref/alt alleles if the AF discordance was less than 15% after swapping (except the palindromic variants). For multi-allelic SNVs and all indels, only variants with the exact ref/alt allele matching and AAF discordance less than 15% were retained. Multi-allelic indels were removed from all imputation results before the comparison.

**Supplementary Method 4. Reference panel simulation and the θ value estimation**

It was unclear how the reference panel affects the θ estimates. To comprehensively investigate the impact of the reference panel on the θ value, we simulated the following five scenarios.

Scenario 1 (Size of a JPT population): We took 100, 500, 1,000, 1,500, 2,000, 2,500, and 3,256 samples from the JPT_3256_. The larger subset always contained all the samples in the smaller subset.

Scenario 2 (Ancestral diversity in a fix-size panel): We extracted 1–5 ancestries from the 1KGP. Then, we shuffled and downsampled the subsets to a size of 504 ten times. We did not maintain consistent ancestry ratios in the downsampled files.

Scenario 3 (Adding large-size EAS samples to a small-size EUR panel): We randomly sampled 403 individuals from the 503 1KGP-EUR samples, combined with the 1KGP-EAS (size = 504) and the six subsets (size = 500–3,256) of the JPT3256 made in Scenario 1.

Scenario 4 (Adding 1KGP samples of distant ancestries to a large-size EAS panel): We combined the JPT3256 with the 1KGP-JPT and 1–5 ancestries in the 1KGP.

Scenario 5 (Simultaneously increase the panel size and ancestral diversity): We extracted 1–5 ancestries from the 1KGP.

Then, we shuffled the samples in each dataset 10 times. Since the dataset had already been cleaned and phased, it was unnecessary to phase it again.

**Supplementary Method 5. Minimac4 source code to obtain the θ value**

In Minimac4’s implementation, the θ value was supplied by either providing it within the m3vcf file or using a reference genetic map [5]. The TOPMed imputation pipeline used the HapMap2 genetic map (see the main article). Minimac4 used linear interpolation to transform the recombination rate to the θ value between markers. By default, the transformed value is an internal variable. We modified the source code to output the values to the log file as follows:

(https://github.com/statgen/Minimac4/blob/v1.0.2/src/Analysis.cpp#L80)

for(i=0; i<referencePanel.numMarkers; i++)

{

std::cout<<"Recom = "<<i<<"\t"<<referencePanel.Recom[i]<<std::endl;

}

**Supplementary Notes**

**Supplementary Note 1. Consistency between MARE and βimp obtained from Rsq and dosage r^2^ (or EmpRsq) and that calculated from imputed dosage and true genotype (or allele)**

In calculating the MARE and βimp metrics, imputed dosage and true genotype (or true allele) were used. When haploid data was used, β_imp_ was the regression slope between the imputed allelic dosage and true allele dose (encoded as 0 or 1), and MARE the residual sum of squares divided by $np\left( 1-p \right)$ (the expected binomial variance on allele), where *n* is the number of haplotypes imputed, and *p* the alternative allele frequency (AAF) in the imputed dataset. When diploid data was used, the linear regression was between the imputed dosage and true genotype (encoded as 0, 1, or 2), β_imp_ was the regression slope, and MARE the residual sum of squares divided by $2np\left( 1-p \right)$ (the expected variance on diploid genotype), where *n* is the number of samples imputed, and *p* the AAF.

As illustrated in the methods and results, three points may break the consistency between MARE and βimp calculated directly from the dosage and obtained from Rsq and dosage r^2^ (or EmpRsq). They were as follows: (1) When dosage r^2^ was calculated from diploid data (when external reference WGS was used) and Rsq from haploid data (Minimac4 reported Rsq in haploid form), Equations 6–7 would suffer from fluctuations caused by violating the Hardy-Weinberg equilibrium (HWE). (2) The AAF inconsistency between the imputed and WGS datasets would affect βimp in Equations 5 and 7. (3) Although Equations 6–7 did not contain AAF, Rsq, and EmpRsq were not stable when the minor allele frequency (MAF) and minor allele count (MAC) were very low [6]. Here, we evaluated each point.

We categorized the variants into two groups according to the MAF of WGS_993_: MAF ≥ 0.5% (MAC ≥ 10) and 0.5% > MAF > 0.1% (10 > MAC > 2). We then calculated the Pearson correlation between (1) AAF in the imputed dataset and WGS_993_, (2) MARE calculated from the imputed dataset and obtained from Rsq and dosage r^2^ (or EmpRsq), and (3) βimp calculated from the imputed dataset and obtained from Rsq and dosage r^2^ (or EmpRsq).

The AAF was highly consistent for variants with MAF ≥ 0.5% (r^2^ > 0.99) but only in modest or weak consistency for variants with 0.5% > MAF > 0.1% (r^2^ = 0.07–0.56) (**Supplementary Figure 3**). The correlation of MARE was > 0.99 and > 0.94 for variants with MAF ≥ 0.5% and with 0.5% > MAF > 0.1% (**Supplementary Figure 4**). Calculating all metrics using the same haploid data fully diminished the fluctuations in MARE, as expected, because AAF discrepancy did not affect Equations 4 and 6 (**Supplementary Figure 5**). The correlation of βimp was > 0.91 and > 0.82 for variants with MAF ≥ 0.5% and with 0.5% > MAF > 0.1% (**Supplementary Figure 6**). It was also in good correlation (> 0.95 for variants with MAF ≥ 0.5%) when calculating from the same haploid data (**Supplementary Figure 7**).

These results showed that Equations 6–7 efficiently predict the estimated MARE and βimp. Thus, on the Rsq ~ dosage r^2^ (or EmpRsq) plot, each region corresponds to specific MARE and βimp values. To reduce fluctuations, we limited all downstream analyses to variants with a MAF ≥ 0.5% and MAC ≥ 10.

**Supplementary Note 2. Explanation of the θ value used in Minimac**

In Minimac’s implementation, there are N hidden states, representing the N reference haplotypes, and M markers, representing the M variants. The θ value is the template switching rate between the adjacent markers. There is a *P = 1 - θ* probability of not switching and a *P = θ* probability of switching to a random reference haplotype, including itself [5,7]. Thus, a low θ value between consecutive markers for a long genome region suggests long stretches of haplotype sharing [8]. The high spikes in the θ value indicate a high probability of recombination events [8]. Li et al. expected that the θ value reflects a combination of population recombination rates and the relatedness between the reference panel and the target samples (MaCH v1.0) [7]. In MaCH, both samples in the reference panel and the target are used to estimate the θ value. Thus, in the current workflow of pre-phasing-imputation, the θ value reveals the intrinsic characteristics of the reference panel; specifically, the similarity of reference haplotypes and the recombination rates [5]. In a typical imputation pipeline of Minimac3-Minimac4, the θ value is estimated from the reference panel using Minimac3 and stored in the m3vcf file. Here, we scaled the θ value manually to study the impacts.

**Supplementary Note 3. Qualification and comparison of the θ value**

Because the θ value is between adjacent markers but not base pairs, the marker density would affect the θ estimates. It is infeasible to directly compare the θ values between large and small panels with different marker densities. However, although the θ values between markers are different, the trend of total θ value along chromosome 19 does not change when constructing different reference panels using the same methods (**Supplementary Figure 16**), as expected, because the θ value reflects the intrinsic property of the reference panel (**Supplementary Note 2**). Hence, we used the total θ value for the comparison.

**Supplementary Note 4. Relationship between imputed-genotype certainty, Rsq, and INFO**

Let *x_i_* be the imputed allelic dosage, also the probability of alternative allele of the *i*-th haplotype, *X_i_*. It has $P\left( X_{i}=1|x_{i} \right)=x_{i}$ and $P\left( X_{i}=0|x_{i} \right)=1-x_{i}$, which means the probability of *X_i_* being 1 is *x_i_*. Given that *X_i_* only takes values 0 or 1, $X_{i}^{2}=\left\{ \begin{aligned} 1, if X_{i}=1 \\ 0, if X_{i}=0 \end{aligned} \right.$, then:

$$E\left[ X_{i}^{2} \right]=x_{i}\times1+\left( 1-x_{i} \right)\times0=x_{i}$$

(Equation 8)

$${E[X_{i}]}^{2}=x_{i}^{2}$$

(Equation 9)

Hence:

$$Var\left[ X_{i} \right]=E\left[ X_{i}^{2} \right]-{E\left[ X_{i} \right]}^{2}=x_{i}-x_{i}^{2}$$

(Equation 10)

$Var\left[ X_{i} \right]$ is the variance of the *i*-th imputed allele. It is 0 when $x_{i}=1 \mathrm{or} 0$, which means no uncertainty. And it is the maximum when $x_{i}=0.5$, which means the true allele has the equal chance to be 0 or 1.

Given *X_i_* is independently imputed for each haplotype, the average variance for all alleles is:

$$\frac{\sum_{i=1}^{2N} Var\left[ X_{i} \right]}{2N}=\frac{\sum_{i=1}^{2N} \left( x_{i}-x_{i}^{2} \right)}{2N}$$

(Equation 11)

where *2N* is the number of imputed haplotypes. Let ***x*** *= (x_1_, ..., x_2N_)* a vector of the imputed allelic dosage and:

$$Var\left( \boldsymbol{x} \right)=\frac{\sum_{i=1}^{2N} x_{i}^{2}}{2N}-{(\frac{\sum_{i=1}^{2N} x_{i}}{2N})}^{2}=\frac{\sum_{i=1}^{2N} x_{i}^{2}-2Np^{2}}{2N}$$

(Equation 12)

where $p=\frac{\sum_{i=1}^{2N} x_{i}}{2N}$ is the alternative allele frequency (AAF).

Add *2Np – 2Np* to the numerator of Equation 12:

$$Var\left( \boldsymbol{x} \right)=\frac{2Np-2Np^{2}-2Np+\sum_{i=1}^{2N} x_{i}^{2}}{2N}$$

$$=\frac{2Np\left( 1-p \right)-\sum_{i=1}^{2N} x_{i}+\sum_{i=1}^{2N} x_{i}^{2}}{2N}$$

$$=p\left( 1-p \right)- \frac{\sum_{i=1}^{2N} \left( x_{i}-x_{i}^{2} \right)}{2N}$$

(Equation 13)

The first term in Equation 13 is the Binomial variance, given all alleles take values 0 or 1 (no uncertainty). The second term is shown in Equation 11. Because:

$$Rsq=\frac{Var(\boldsymbol{x})}{p(1-p)}=1-\frac{\sum_{i=1}^{2N} \left( x_{i}-x_{i}^{2} \right)}{2Np(1-p)}$$

(Equation 14)

the inverse relationship between Rsq and $Var\left[ X_{i} \right]$ is thereby demonstrated. When all *x_i_* = *p*, there is no information gain from imputation, and Rsq = 0, as expected. When all *x_i_* take values 0 or 1, $x_{i}-x_{i}^{2}=0$, and Rsq = 1, also as expected.

Next, we demonstrate that Rsq equals INFO score. INFO is defined as [9]:

$$INFO=1-\frac{\sum_{j=1}^{M} \left( 4P_{jg2}+P_{jg1}-\left( x_{ja}+x_{jb} \right)^{2} \right)}{2Mp\left( 1-p \right)}$$

(Equation 15)

Where *j* is the *j*-th individual and *ja* *jb* the imputed dosage of two alleles. *P_jg1_* and *P_jg2_* are probabilities of the imputed genotype being 1 or 2 for individual *j*. *M* is the number of individuals, and *p* the AAF. Given *ja* and *jb* are independent, $P_{jg1}=x_{ja}\left( 1-x_{jb} \right)+(1-x_{ja})x_{jb}$ and $P_{jg2}=x_{ja}x_{jb}$. Then:

$$INFO=1-\frac{\sum_{j=1}^{M} (4x_{ja}x_{jb}+x_{ja}\left( 1-x_{jb} \right)+\left( 1-x_{ja} \right)x_{jb}-\left( x_{ja}+x_{jb} \right)^{2})}{2Mp\left( 1-p \right)}$$

$$=1-\frac{\sum_{j=1}^{M} (x_{ja}-x_{ja}^{2}+x_{jb}-x_{jb}^{2})}{2Mp(1-p)}$$

(Equation 16)

In the same dataset, *(x_1a_, x_1b_, ..., x_Ma_, x_Mb_) = (x_i_, …, x_2N_*) and *M = N*, thus we have:

$$\sum_{j=1}^{M} \left( x_{ja}-x_{ja}^{2}+x_{jb}-x_{jb}^{2} \right)=\sum_{i=1}^{2N} \left( x_{i}-x_{i}^{2} \right)$$

(Equation 17)

Finally, for a variant:

$$INFO=1-\frac{\sum_{i=1}^{2N} \left( x_{i}-x_{i}^{2} \right)}{2Np\left( 1-p \right)}=Rsq$$

(Equation 18)

Taking Equations 11 and 18 together, Rsq and INFO are equal when calculating from the same dataset, and inversely associated with the average variance of imputed allele. High certainty of an imputed allele decreases the variance and thereby increases Rsq and INFO.

**Supplementary Note 5. Selection criteria for rs142572000 and rs671 and three real-world examples**

We set two extreme cases to compare the imputed-dosage distribution: (1) similar Rsq but different dosage r^2^: difference in Rsq < 0.05 and difference in dosage r^2^ > 0.3; (2) similar dosage r^2^ but different Rsq: difference in dosage r^2^ < 0.05 and difference in Rsq > 0.1, between the TOPMed and JEWEL3k imputation results. Rs142572000 is a random example of case (1). It is an EAS-specific low-frequency variant; the MAF in 8.3k JPN (WGS of 8300 Japanese) is 0.059 and that in TOPMed is 0.000064 (both MAF values were retrieved from dbSNP). On the other hand, rs671 is a specific variant fulfilling case (2). The average array marker density is 0.0077 (array marker/imputed marker: 25347/3276841) for chr12, while it is 0.0028 (20/7060) and 0.0025 (40/16082) for the nearest 20 and 40 markers surrounding rs671. In general, the low-density array has been reported to create high uncertainty and it was also replicated in the JEWEL3k imputation result (**Figure 3J**) [10]; however, TOPMed imputation seemed to be robust to the density (**Figure 3G**), as reported in the literature [11]. Our analysis showed that for the JEWEL3k WGS, the TOPMed imputation pipeline used a 0.267-fold θ value, compared with the Minimac3-Minimac4 pipeline we used (**Supplementary Table 10**). The higher θ value and low array marker density at this region caused the lower Rsq of rs671 in the JEWEL3k imputation result.

To judge whether observations in **Figure 3** were specific to the selected variants, we examined the imputed dosage of variants reported in GWAS. For case (1), we raised other examples that showed a similar Rsq (difference < 0.05) and different dosage r^2^ (difference > 0.1) between TOPMed and JEWEL3k imputation results (11,770 variants on chr19 passed the filter). We annotated these variants using <https://www.snp-nexus.org/v4> (annotation: NHGRI Catalogue of Published Genome-Wide Association Studies) [12], and 70 variants were associated with at least one trait. Then, three variants (rs1047781, rs113230003, and 76246107) with the most reports were selected to show the imputed dosage against the true genotype (**Supplementary Table 3** and **Supplementary Figure 8**).

For rs10477781, using both panels, Rsq was higher than 0.82. However, dosage r^2^ was 0.774 and 0.890 in TOPMed and JEWEL3k imputation results, respectively. As shown in **Supplementary Figure 8**, in the TOPMed imputation result, heterozygotes were imputed as homozygotes, and alternative allele homozygotes were imputed as heterozygotes. Although the majority of individuals were correctly imputed (as shown by the IQR), these wrongly imputed genotypes increased MARE and decreased the correlation coefficient.

For rs113230003, Rsq was 0.883 and 0.925 in the TOPMed and JEWEL3k imputation results, and dosage r^2^ was 0.847 and 0.950, respectively. In both results, Rsq roughly equaled dosage r^2^. JEWEL3k created fewer wrongly imputed genotypes and narrower IQR, leading to higher Rsq, β_imp_, and dosage r^2^, and lower MARE.

For rs76246107, the two results showed different patterns. In the TOPMed result, Rsq (0.681) > dosage r^2^ (0.531). Heterozygotes were imputed as homozygotes, and the overall imputed dosages were shrunk to the mean, leading to high MARE (0.583) and low β_imp_ (0.315). In the JEWEL3k result, Rsq (0.730) < dosage r^2^ (0.807). The imputed dosages were shrunk to the mean (β_imp_ = 0.757), while the IQRs were narrower than those for the TOPMed result; fewer wrongly imputed genotypes were observed (MARE = 0.139). This example indicated that the imputed dosage impacted the four metrics simultaneously.

Similar to rs142572000 and rs671, the imputation errors (*e.g.*, imputed the homozygote as heterozygote and vice versa) would cause higher MARE and lead to Rsq > dosage r^2^; shrinkage in the imputed dosage would cause lower β_imp_ and lead to Rsq < dosage r^2^. Moreover, for these variants with similar Rsq (difference < 0.05) in TOPMed and JEWEL3k results, the TOPMed imputation result was of higher certainty but contained more imputation errors.

For case (2), rs671 is a well-known functional variant associated with alcohol metabolism. Because the JEWEL3k panel was population-specific, its imputation result naturally outperformed TOPMed in dosage r^2^. Using the criteria of (1) difference in dosage r^2^ < 0.05; (2) difference in Rsq > 0.1, no variant on chr19 was left. Thus, we did not raise other examples.

We further evaluate the imputed-genotype certainty of all variants on chr19. Rsq and the imputed genotype certainty are positively related, as shown in our analytical derivation (**Supplementary Note 4**). However, each variant has a different imputed-genotype certainty level, making it difficult to generalize the trend between the deviation (Rsq – dosage r^2^) and imputed-genotype certainty. Here, we compared the same variants between the TOPMed and JEWEL3k imputation results, where JEWEL3k imputation was used for the deviation-free control. We defined a certainly imputed genotype if the imputed genotype probability (GP) for any genotypes (0/0, 0/1, or 1/1) is above 0.9. We grouped the variants in each dosage r^2^ bin into four groups according to the deviation (Rsq – dosage r^2^) in the TOPMed imputation result: [-1, -0.1), [-0.1, 0), [0, 0.1), and [0.1, 1]. We extracted these variants from the JEWEL3k imputation result. From both datasets, we calculated the mean deviation (Rsq – dosage r^2^) and its 95% CI from bootstrapping (1,000 times). In the JEWEL3k imputation result, we did not observe Rsq > dosage r^2^ for all the dosage r^2^ bins and Rsq – dosage r^2^ groups (**Supplementary Table 4**), indicating JEWEL3k is a valid control group. Then, we evaluated relative imputed-genotype certainty in TOPMed imputation compared to JEWEL3k imputation by the ratio of the number of imputed-genotypes with GP > 0.9 (TOPMed/JEWEL3k). In dosage r^2^ bins [0.6, 1], the relative imputed-genotype certainty increased as the deviation (Rsq - dosage r^2^) increased (**Supplementary Table 4**). Thus, we conclude that, in many cases, variants with higher Rsq in the same dosage r^2^ bin have higher relative imputed-genotype certainty, compared to the same variants in the JEWEL3k imputation result.

**Supplementary Note 6. Selection criteria for rs10410162 and three real-world examples**

To show how the four metric values change with the θ value, we set the following criteria: (1) better distinguishability between the imputed allelic dosage of the two alleles (EmpRsq > 0.6, MAF > 0.1, |Rsq – EmpRsq| < 0.1, and β_imp_ > 0.7 when using the 1-fold θ value); (2) better resolution of the changes (the |Rsq – EmpRsq | > 0.2, when using the 0.01-fold θ value); (3) avoiding potential bias from the margin of the chromosome (between the 10 Mb and 45 Mb position on chromosome 19). Rs10410162 was randomly selected as an example.

We used the same variants in **Supplementary Note 5**, rs1047781, rs113230003, and rs76246107, as real-world examples to enhance the evidence. Metric values and imputed dosage distributions were shown in **Supplementary Table 5** and **Supplementary Figure 12**. Downscaling of the θ value had shrunk the imputed dosages to 0, 1, or 2 regardless of the true genotype, and finally led to Rsq > dosage r^2^. Upscaling of the θ value had shrunk all imputed dosages to the panel mean and decreased the β_imp_ and Rsq, but dosage r^2^ could hold for a range of θ values.

To enhance the evidence, we quantified the trend of deviation (Rsq – EmpRsq) and the imputed-allele certainty (the number of confident alleles; HDS > 0.9) for the array markers (MAF ≥ 0.005, 10,273 variants), and the trend of the imputed-allele certainty for all imputed markers (1,084,535 variants; EmpRsq is not available). In these analyses, we used bootstrapping to evaluate the 95% confidence intervals (95% CIs) of these observations for each θ value. In short, the trends were concordant with our previous descriptions: When we increase θ value from 0.01- to 100-fold, for array markers, the mean of deviation (Rsq – EmpRsq) decreased from 0.063 to -0.343, the mean Rsq decreased from 0.924 to 0.083, and the number of confident alleles decreased from 7.04 million to 859,124. The highest mean EmpRsq (created by the 0.8-fold θ value) showed overlapping 95% CI with that created from 0.1- to 2-fold θ values, indicating EmpRsq is insensitive to a range of θ values (**Supplementary Table 6**). When we increase θ value from 0.01- to 100-fold, for all imputed markers, the mean Rsq decreased from 0.281 to 0.052 and the number of confident alleles decreased from 111.90 million to 16.30 million. These results were concordant with that listed in the manuscript.

**Supplementary Note 7. Explanation of the change in the θ value and the imputed allelic dosage**

We found that different θ values changed the imputed allelic dosage. The imputation aims to find the unobserved paths through hidden states (corresponding to the reference haplotypes) to build an imperfect mosaic of reference haplotypes that matches the target haplotype while allowing for allele mismatches [6]. The template switching rate (θ) is the probability that the path switches from one state to the next. [5]The error rate (ε) represents the tolerance of mismatches between the selected mosaic path and the target haplotype. [5]The posterior probability of the imputed allele is the state probabilities weighted sum of the allele on each reference haplotype corresponding to that hidden state (for the missing markers, the emission probability of the state is 1) [6].

When running the imputation, only genotyped markers are available to find the paths. Switching and error rates could be interpreted as how easier a switch would happen and how many mismatches are acceptable. Given a specific ε value, switches are suppressed if all markers have θ values close to 0. The imputation algorithm would copy the most similar reference haplotype (*i.e.*, with the least mismatches) rather than finding a mosaic path. It would make the imputation result with less or no uncertainty (*i.e.*, all the imputed allelic dosages are 0 or 1 as that of the selected reference haplotype) but with more wrongly imputed alleles [13], because this process does not always select haplotype(s) from the same ancestor (0.01–0.5 fold scaling of the θ value in **Supplementary Figure 14**). Conversely, if all markers have high θ values, many mosaic paths which could produce fewer errors in the genotyped markers are selected [13]. [10]On average, it would shrink the imputed allelic dosage of missing markers to the reference panel’s mean (2–100-fold scaling of the θ value in **Supplementary Figure 14**). If a marker has a higher ε value, then this marker is less weighted when computing the posterior probability of the path. In that case, all states obtain a similar posterior probability, regardless of whether the allele is matched or mismatched between the reference and target haplotype. Here, we studied the θ value.

**Supplementary Note 8. Reference panel’s impact on the θ estimates**

**Figure 6** conveys five crucial findings regarding the influence of reference panel composition on the θ estimates.

First, the total θ value decreased consistently (p = 7.85e-5 for each size increase, Wilcoxon rank-sum test, one-sided), with the trend being more modest as the JPT-only panel size increased (**Figure 6A**). This suggested that when the reference panel size was small (*e.g.*, < 2,000), and additional samples made it easier to find shared haplotypes, hence expecting fewer switches. On the other hand, at a larger size, the plentiful haplotype sharing might lead to further additions of samples with modest effects.

Second, when the panel size was fixed and only changed the ancestral composition, the total θ value decreased when mixing EUR with EAS (p=5.04e-4, Wilcoxon rank-sum test, one-sided) and increased with further mixing AFR (p=7.85e-5, same as above), AMR (p=0.298, same as above), and SAS (p=7.85e-5, same as above) (**Figure 6B**). The ancestral diversity might make it harder to find shared haplotypes.

Third, in a EUR-EAS multi-ancestry panel, with the panel size increased from 403 to 4,163 and the proportion of EUR samples decreased from 100% to 9.68%, the total θ value decreased from 741.3 to 385.6, with a decrease of 52.0% (p=9.70e-4, 7.85e-5, 7.85e-5, 2.53e-4, 1.91e-4, 7.85e-5, 1.25e-5, for each size increase, Wilcoxon rank-sum test, one-sided) (**Figure 6C**). The ancestral diversity was more unbalanced in the large multi-ancestry reference panels. For example, the percentages of EAS samples in the HRC and TOPMed panels were 1.55% and 1.22%, respectively. Based on our simulation results, these reference panels might produce even lower θ value estimations than that estimated from an EAS-only reference panel with comparative sizes of EAS samples in those large multi-ancestry panels.

Fourth, adding 1KGP-JPT to the JPT3256 slightly increased the total θ value (an increment of 1.2%; p=0.013, Wilcoxon rank-sum test, one-sided) (**Figure 6D**). The total θ value increased with further addition of the remaining samples of 1KGP-EAS (p=1.43e-4, same as above), EUR (p=1.43e-4, same as above), AFR (p=1.91e-4, same as above), AMR (p=0.128, same as above), and SAS (p=6.31e-3, same as above). However, the total θ value changed from 327.7 to 443.8, with an increment of 35.4%, from JPT3256 to JPT3256+1KGP, revealing that even if the major ancestry group comprised only 65.28% of the samples in the reference panel, the change in the total θ value was relatively moderate.

Fifth, first with 104 1KGP-JPT, the total θ value decreased with the addition of the remaining EAS (p=7.85e-5, Wilcoxon rank-sum test, one-sided), EUR (p=7.85e-5, same as above), AFR (p=7.85e-5, same as above), and AMR (p=7.85e-5, same as above) (**Figure 6E**). Thus, the total θ value decreased as the sample size increased, regardless of the ancestry added, for the small panel size. Subsequently, the total θ value stopped decreasing at a sample size of approximately 2,000 with four ancestries. Further expanding to the five ancestries of 1KGP almost never changed the total θ value (p=0.910, Wilcoxon rank-sum test, two-sided). Combined with the simulation results from **Figures 6A–B**, the total θ value was in a trade-off between the panel size and ancestral diversity.

In summary, the θ estimates decreased with the sample size of a single ancestry and increased with ancestral diversity when size was fixed. While simultaneously increasing the panel size and ancestral diversity, the θ estimates were in a trade-off. The result also suggested that in a multi-ancestry panel of more than several thousand subjects, the θ estimates were underestimated for the minor ancestry and overestimated for the major ancestry compared to the value estimated in the single-ancestry panel.

**Supplementary Note 9. Replication using SNPs on chromosome 20**

To assess the reliability of our conclusions, we replicated the simulation analyses using SNPs on chr20 (Indels were removed). Specifically, we replicated: (1) manual scaling of the θ value in the 1KGP reference panel; (2) the θ value estimation using reference panels with different sizes and ancestral diversities; (3) imputation of a major (JPT) and a minor (EUR) ancestry using multi-ancestry reference panels.

For (1), downscaling of the θ value increased Rsq, but not EmpRsq (a difference less than 0.03 for the θ value between 0.01- and 2-fold), thus causing Rsq > EmpRsq (**Supplementary Table 11**); 4.93% more variants reached Rsq > 0.7 when the θ scaling changed from 1- to 0.5-fold (**Supplementary Table 12**). The number of variants with Rsq > 0.7 decreased by 6.67% from 1- to 2-fold θ value. On the other hand, upscaling of the θ value decreased Rsq but not EmpRsq, unless it was scaled up to 5-fold (a maximum difference of 0.055 between 1- and 5-fold θ value). This result replicated that EmpRsq was insensitive to altering the θ value, particularly the downscaling, while Rsq and the number of high-Rsq variants increased with the θ value downscaling.

For (2), as shown in **Supplementary Table 13–16**, the size of a single ancestry in the reference panel decreased the θ value estimation, while the ancestral diversity increased it.

For (3), as shown in **Supplementary Table 17**, when setting 403 EUR as the minor ancestry, with more EAS samples added to the reference panel, Rsq increased, while EmpRsq slightly decreased (a maximum difference of 0.033). In the imputation result, 791 of the 172,228 variants (0.46%) that reached Rsq > 0.7 were EAS-specific (**Supplementary Table 18**), even if the proportion of EAS samples in the reference panel was 90.32%. When setting EAS as the major ancestry, with higher ancestral diversity in the reference panel, Rsq decreased (a decrease of 0.005, 0.019, and 0.034 for variants with MAF ≥ 5%, 1% ≤ MAF < 5%, and 0.05% ≤ MAF < 1%, respectively, from 3256JPT+1KGP-EAS to 3256JPT+1KGP), while EmpRsq slightly decreased (a maximum difference of 0.003) (**Supplementary Table 19**). 2.61% fewer variants reached Rsq > 0.7 from 3256JPT+1KGP-EAS to 3256JPT+1KGP, and non-EAS variants only comprised 0.23% of the total amount of variants in 3256JPT+1KGP (**Supplementary Table 20**).

In summary, all conclusions in the main manuscript held in the replication (see Conclusion).

**Supplementary Note 10. Number of confident alleles and high-Rsq variants obtained from distant ancestries using the 1KGP panel**

We showed that no matter the target sample was the minor or major ancestry of the multi-ancestry reference panel, only a few variants existing on the haplotypes of distant ancestry could pass Rsq > 0.7. Thus, we concluded that we would not expect to gain a significant number of high-Rsq variants (Rsq > 0.7) when combining the distant ancestry to expand the reference panel size. In scenarios 1 and 2 of the main article, we merged the JPT WGS and the 1KGP datasets by IMPUTE2. It has been reported that using IMPUTE2 to merge panels may bring variants from one panel to the missing sites of another panel [14]. Hence, our methods may underestimate the number of high-Rsq variants from each ancestry (in case a EUR variant is first imputed to the JPT reference haplotype and then to the target sample, it would neither be counted as EUR-only nor JPT-only).

To further quantify the contribution of high-Rsq variants from haplotypes of distant ancestry, we used the 1KGP panel. We defined the EAS-only and non-EAS variants straightforwardly. EAS-only variants only existed in the 504 EAS samples and non-EAS variants only existed in the 2,000 non-EAS samples. There were 83,023 EAS-only and 696,396 non-EAS variants. Using WGS_993_ as the target sample, the number of confident alleles (HDS > 0.9) was 98.9M (4.59% of the total variants × individual pairs), in which 185,870 or 3,362 belonged to EAS-only or non-EAS variants (the 1-fold θ value in **Supplementary Table 7**), respectively. Among the 159,469 high-Rsq variants (14.7% of the total variants), 8,627 and 724 were EAS-only or non-EAS, corresponding to a ratio of 10.4% and 0.10% of the total EAS-only and non-EAS variants, respectively (**Supplementary Table 7**). The result verified our conclusion that non-EAS variants were not rigorously presented in the imputation results.

**Supplementary Figures**

**Supplementary Figure 1. Dosage r^2^ of all imputed variants.**

The box plot shows dosage r^2^ of all imputed SNVs (A) and indels (B) (with Rsq ≥ 0.3 and in WGS_993_) using the TOPMed, 1KGP, BBJ1k, and JEWEL3k reference panels, stratified by MAF of WGS_993_. The boxes show the median, upper (75%), and lower (25%) quartiles. The whiskers show the 1.5-fold interquartile range (IQR) extended from the upper or lower quartile if a value exceeds them; otherwise, show the maximum or minimum value.

**Supplementary Figure 2. Comparison of dosage r^2^ and Rsq in each MAF bin.**

The figure shows the deviation between dosage r^2^ (x-axis) and Rsq (y-axis) in the TOPMed (A), 1KGP (B), BBJ1k (C), and JEWEL3k (D) imputation results. The scatter represents the variants, with the density shown by the contour lines. The diagonal line shows that Rsq equals dosage r^2^, and the histograms on the side show the distribution. Overlapping variants imputed by the four panels were used. MAF is determined by WGS_993_.

**Supplementary Figure 3. Comparison between the alternative allele frequency (AAF) of the imputed and WGS datasets.**

The scatter plot shows the comparison between the AAF of the imputation result and WGS_993_. Variants were grouped into (A) MAF ≥ 0.5% and (B) 0.5% > MAF ≥ 0.1% by MAF of WGS_993_. In (B), variants with both 0.5% > MAF ≥ 0.1% and AAF < 0.5% are used.

**Supplementary Figure 4. Comparison between MARE calculated from imputed dosage and obtained from Rsq and dosage r^2^.**

The scatter plot shows the comparison between MARE calculated from imputation results or obtained from Rsq and dosage r^2^. Variants were grouped into (A) MAF ≥ 0.5% and (B) 0.5% > MAF ≥ 0.1% by MAF of WGS_993_.

**Supplementary Figure 5. Comparison between MARE calculated from imputed allelic dosage and obtained from Rsq and EmpRsq.**

The scatter plot shows the comparison between MARE calculated from imputed allelic dosage (LooDosage) or obtained from Rsq and EmpRsq. LooDosage and EmpRsq are from the leave-one-out imputation of Minimac4 by hiding markers on the genotyping array. Variants were grouped into (A) MAF ≥ 0.5% and (B) 0.5% > MAF ≥ 0.1% by MAF of the genotyping array.

**Supplementary Figure 6. Comparison between β_imp_ calculated from imputed dosage and obtained from Rsq and dosage r^2^.**

The scatter plot shows the comparison between β_imp_ calculated from imputation results or obtained from Rsq and dosage r^2^. Variants were grouped into (A) MAF ≥ 0.5% and (B) 0.5% > MAF ≥ 0.1% by MAF of WGS_993_.

**Supplementary Figure 7. Comparison between β_imp_ calculated from imputed allelic dosage and obtained from Rsq and EmpRsq.**

The scatter plot shows the comparison between β_imp_ calculated from imputed allelic dosage (LooDosage) or obtained from Rsq and EmpRsq. Variants were grouped into (A) MAF ≥ 0.5% and (B) 0.5% > MAF ≥ 0.1% by MAF of the genotyping array.


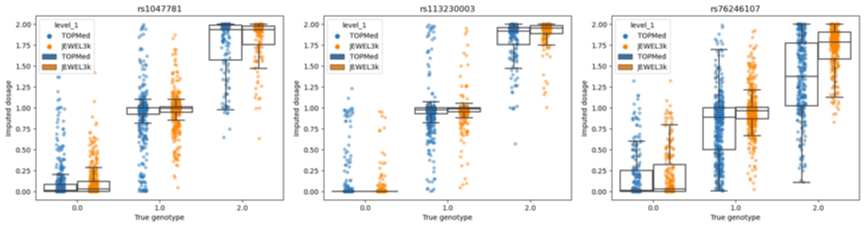


**Supplementary Figure 8. Comparison between the imputed dosage and the true genotype of rs1047781, rs113230003, and rs7624610 in the TOPMed and JEWEL3k imputation results.**

The figure shows the imputed dosage of rs1047781, rs113230003, and rs7624610 in the imputation results using the TOPMed and JEWEL3k reference panels. The strip plots show the imputed dosage, and the true genotype is determined by WGS. The boxes show the median, upper (75%), and lower (25%) quartiles. The whiskers show the 1.5-fold interquartile range (IQR) extended from the upper or lower quartile if a value exceeds them; otherwise, show the maximum or minimum value.

**Supplementary Figure 9. MARE and β_imp_ of variants on chr19 using the TOPMed, 1KGP, BBJ1k, and JEWEL3k reference panels.**

(A) The mean MARE stratified by Rsq in each imputation result. (B) The mean β_imp_ stratified by dosage r^2^ in each imputation result. In (A) and (B), each bin has a width of 0.05, and bins with less than 50 variants are not shown. The expected values are calculated by assuming that Rsq equals dosage r^2^. Overlapping variants imputed by the four panels were used. MAF is determined by WGS_993_ and variants with MAF ≥ 0.5% were used.


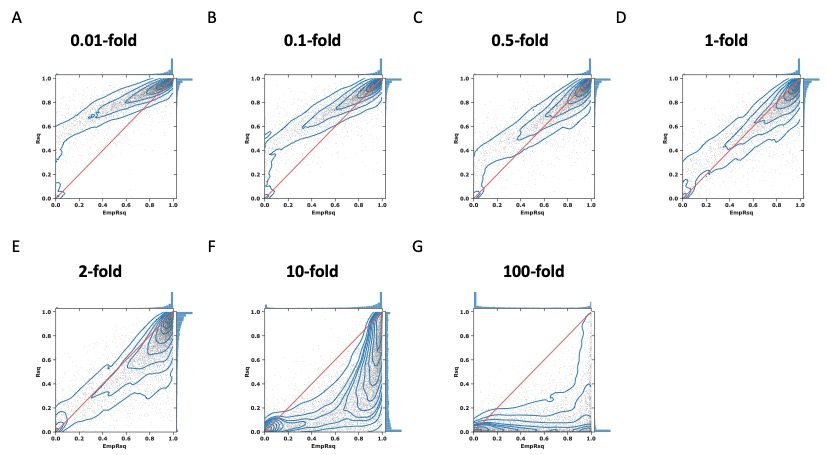


**Supplementary Figure 10. Comparison of EmpRsq and Rsq in the imputation results using different scalings of the θ value.**

The figure shows the deviation between EmpRsq (x-axis) and Rsq (y-axis) in the imputation results using the 1KGP reference panel, WGS_993_ as the target sample, and seven scalings of the θ value. (A) 0.01-fold; (B) 0.1-fold; (C) 0.5-fold; (D) 1-fold (original); (E) 2-fold; (F) 10-fold; (G) 100-fold. The scatter represents the variants, with the density shown by the contour lines. The diagonal line shows that Rsq equals EmpRsq, and the histograms on the side show the distribution.

**Supplementary Figure 11. Allelic dosage distribution of rs1041062 in the imputation results using different scalings of the θ value.**

The figure shows the imputed allelic dosage of rs1041062 in the imputation results using the 1KGP reference panel, WGS_993_ as the target sample, and seven scalings of the θ value. (A) 0.01-fold; (B) 0.1-fold; (C) 0.5-fold; (D) 1-fold (original); (E) 2-fold; (F) 10-fold; (G) 100-fold. The strip plots show the imputed allelic dosage and the violin plots show the distribution. The regression line is between the imputed allelic dosage and the true allele.


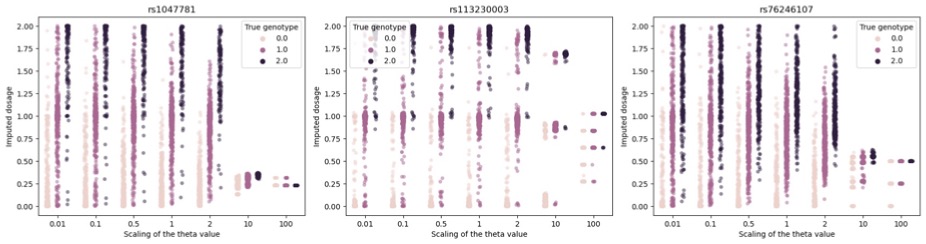


**Supplementary Figure 12. Comparison between the imputed dosage and the true genotype of rs1047781, rs113230003, and rs7624610 in the imputation results using different scalings of the θ value.**

The figure shows the imputed dosage of rs1047781, rs113230003, and rs7624610 in the imputation results using the 1KGP reference panel, WGS_993_ as the target sample, and seven scalings of the θ value. The strip plots show the imputed dosage.


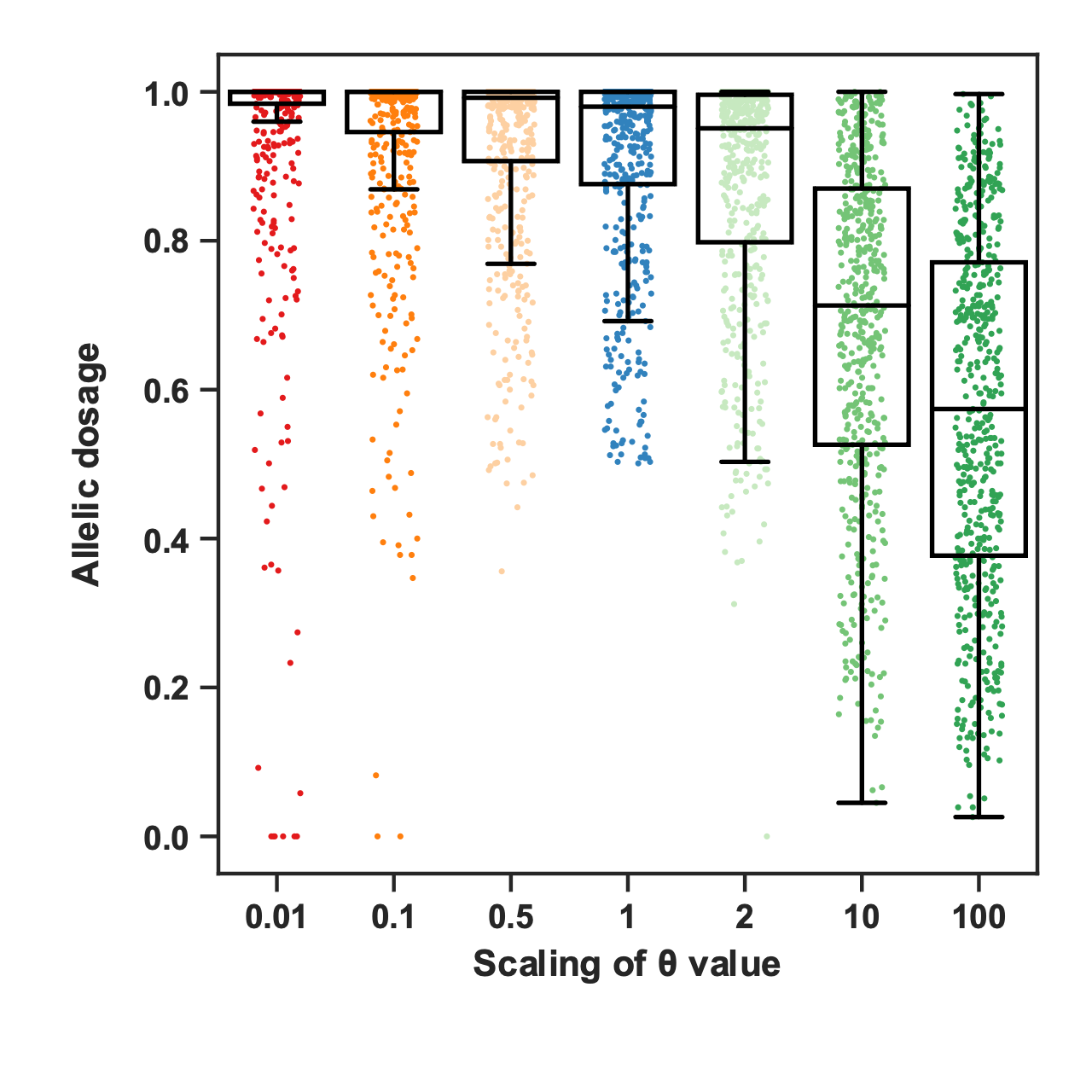


**Supplementary Figure 13. Distribution of the imputed allelic dosage with different scalings of the θ value.**

The figure shows the imputed allelic dosage of a randomly selected target haplotype (the same as Figure 4D) with the 7 scalings of the θ value. The scatters show variants. The boxes show the median, upper (75%), and lower (25%) quartiles. The whiskers show the 1.5-fold interquartile range (IQR) extended from the upper or lower quartile if a value exceeds them; otherwise, show the maximum or minimum value. Only variants with imputed allelic dosage > 0.5 and 0.3 < EmpRsq < 0.8 when using the 1-fold θ value are shown.

**Supplementary Figure 14. Changes in the imputed allelic dosage with different scalings of the θ value.**

The figure shows the changes in the imputed allelic dosage for the same target haplotype used in Figure 4D (A) and Supplementary Figure 13 (B). Each line represents a variant, the x-axis represents the scaling and the y-axis represents the imputed allelic dosage. A lower θ value only shrinks the dosages to 0 or 1 (increase certainty) but does not guarantee accuracy. On the other side, a higher θ value shrinks all imputed dosages to 0.5 (increase uncertainty).

**Supplementary Figure 15. MARE and β_imp_ of variants on chr19 using the 1KGP reference panel and different scalings of the θ value.**

(A) The mean MARE stratified by Rsq in each imputation result. (B) The mean β_imp_ stratified by EmpRsq in each imputation result. In (A) and (B), each bin has a width of 0.05, and bins with less than 50 variants are not shown. The expected values are calculated by assuming that Rsq equals EmpRsq. MAF is determined by the genotyping array and variants with MAF ≥ 0.5% were used.


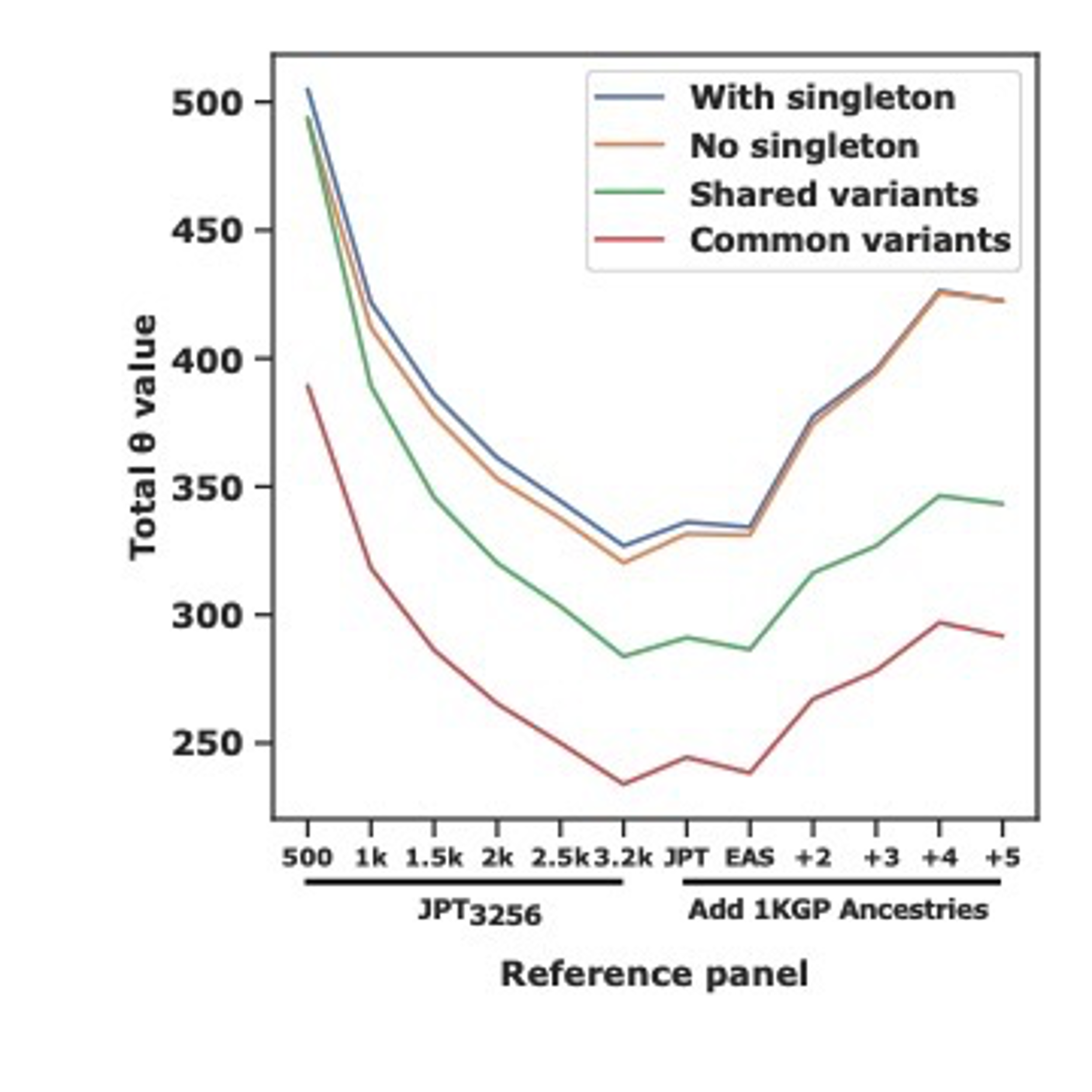


**Supplementary Figure 16. Total θ value along chromosome 19.**

The figure shows the total θ value along chromosome 19 estimated by Minimac3. Four different panel construction methods were compared. The x-axis labels are 500–3.2k: the JPT_3256_ subset size; “JPT”, EAS”, and “+2”–“+5” ancestries: the 3,256 JPT + 1KGP-JPT, 1KGP-EAS, and the other 2–5 ancestries in the 1KGP, in the order of EUR, AFR, AMR, SAS. Panel construction methods are “With singleton”: monomorphic variants and singletons are not removed when making subsets. These panels contain the same markers (identical number and position), with a size = 1.53M. “No singleton”: monomorphic variants and singletons are removed. Number of markers in these panels varies from 510k to 1.53M. This is the routine strategy to build a reference panel for Minimac3/4. “Shared variants”: all panels contain identical variants set, with a size of 370k (no singletons). “Common variants”: each panel only contains variants with MAF ≥ 5%. MAF is determined by samples of each panel.


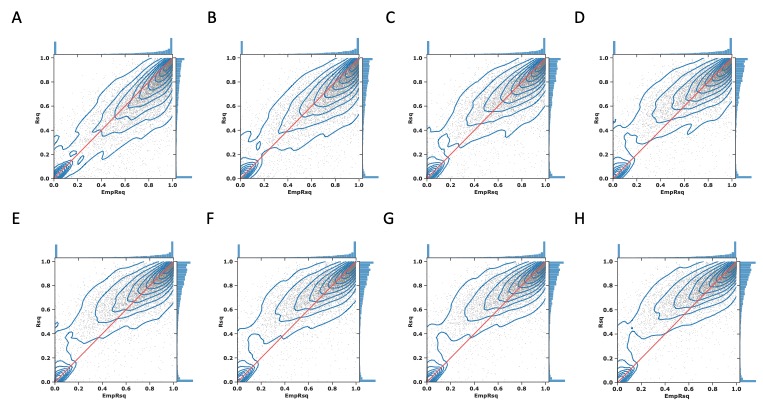


**Supplementary Figure 17. Comparison of EmpRsq and Rsq in the imputation results using simulated EUR-EAS reference panels.**

The figure shows the deviation between EmpRsq (x-axis) and Rsq (y-axis) in the imputation results using 8 simulated EUR-EAS reference panels. (A) EURn403; (B) EURn403 +1KGP-EAS; (C) EURn403 +1KGP-EAS+500JPT; (D) EURn403 +1KGP-EAS+1000JPT; (E) EURn403 +1KGP-EAS+1500JPT; (F) EURn403 +1KGP-EAS+2000JPT; (G) EURn403 +1KGP-EAS+2500JPT; (H) EURn403 +1KGP-EAS+3256JPT. The scatter represents the variants, with the density shown by the contour lines. The diagonal line shows that Rsq equals EmpRsq, and the histograms on the side show the distribution. EURn403 represents the 403 EUR; 500–3256JPT represents the number of JPT samples in the panel.

**Supplementary Figure 18. MARE and β_imp_ of variants on chr19 using the simulated EUR-EAS reference panels.**

(A) The mean MARE stratified by Rsq in each imputation result. (B) The mean β_imp_ stratified by EmpRsq in each imputation result. In (A) and (B), each bin has a width of 0.05, and bins with less than 50 variants are not shown. The expected values are calculated by assuming that Rsq equals EmpRsq. MAF is determined by the array and variants with MAF ≥ 0.5% were used. EURn403 represents the 403 EUR; 500–3256JPT represents the number of JPT samples in the panel.


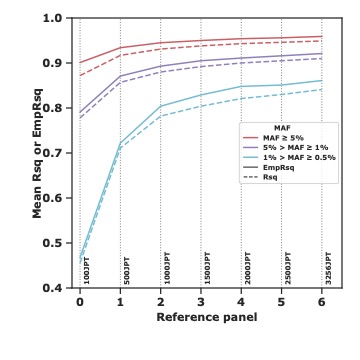


**Supplementary Figure 19. Mean EmpRsq and Rsq using JPT-only reference panels with different sizes.**

The plot shows the mean Rsq and EmpRsq of each imputation result, stratified by MAF of the array. The x-axis shows the reference panel indexes. Reference panels are indicated by the vertical dashed lines and labeled. 500–3256JPT represents the number of JPT samples in the panel.

**Supplementary Figure 20. MARE and β_imp_ of variants on hr19 using the 7 simulated JPT-1KGP reference panels.**

(A) The mean MARE stratified by Rsq in each imputation result. (B) The mean β_imp_ stratified by EmpRsq in each imputation result. Each bin has a width of 0.05, and bins with less than 50 variants are not shown. The expected values are calculated by assuming that Rsq is equal to EmpRsq. MAF is determined by the array and variants with MAF ≥ 0.5% were used.


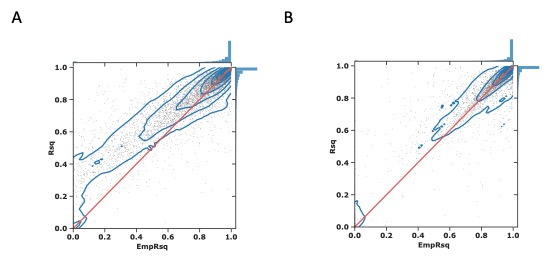


**Supplementary Figure 21. Comparison of the EmpRsq and Rsq in the imputation results using the HapMap2 genetic map as a reference for the θ value.**

The figure shows the deviation between EmpRsq and Rsq in the imputation results using the Minimac4 v1.0.2 and HapMap2 genetic map, the 1KGP (A) and JEWEL3k (B) as the reference panel, and WGS_993_ as the target sample. The scatter represents the variants, with the density shown by the contour lines. The diagonal line shows that Rsq equals EmpRsq, and the histograms on the side show the distribution.


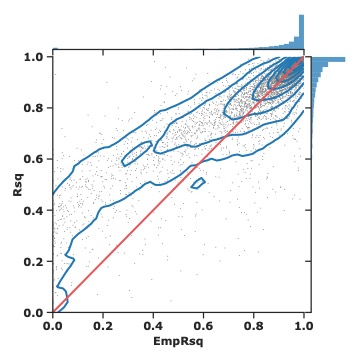


**Supplementary Figure 22. Comparison of the EmpRsq and Rsq in the imputation results using the HRC reference panel.**

The figure shows the deviation between EmpRsq and Rsq in the imputation results using the HRC reference panel (performed on the Michigan Imputation Server). The scatter represents the variants (on chr22), with the density shown by the contour lines. The diagonal line shows that Rsq equals EmpRsq, and the histograms on the side show the distribution.

**Supplementary Tables**

**Supplementary Table 1. Number of imputed variants using the four reference panels.**

| Reference panel | MAF (%) | SNV | | | Indel | | | All |
| --- | --- | --- | --- | --- | --- | --- | --- | --- |
|  |  | Rsq ≥ 0 | Rsq ≥ 0.3 | Rsq ≥ 0.7 | Rsq ≥ 0 | Rsq ≥ 0.3 | Rsq ≥ 0.7 | Rsq ≥ 0 |
| TOPMed | [0, 0.05) | 252,771,622 | 7,573,456 | 1,218,495 | 19,491,389 | 553,114 | 88,286 | 272,263,011 |
|  | [0.05, 0.5) | 8,592,816 | 5,424,023 | 2,182,279 | 681,658 | 434,383 | 169,287 | 9,274,474 |
|  | [0.5, 1) | 1,313,057 | 1,075,612 | 738,434 | 104,566 | 87,431 | 58,194 | 1,417,623 |
|  | [1, 5) | 2,324,580 | 1,979,385 | 1,633,050 | 176,655 | 156,127 | 126,582 | 2,501,235 |
|  | [5, 50] | 5,387,174 | 5,339,524 | 5,150,778 | 370,890 | 368,148 | 354,359 | 5,758,064 |
|  | All | 270,389,249 | 21,392,000 | 10,923,036 | 20,825,158 | 1,599,203 | 796,708 | 291,214,407 |
| 1KGP | [0, 0.05) | 26,375,615 | 913,226 | 185,101 | 1,484,317 | 60,437 | 13,708 | 27,859,932 |
|  | [0.05, 0.5) | 8,201,495 | 2,075,814 | 502,547 | 601,000 | 149,797 | 34,227 | 8,802,495 |
|  | [0.5, 1) | 1,262,622 | 803,635 | 357,303 | 112,610 | 70,492 | 26,261 | 1,375,232 |
|  | [1, 5) | 2,318,671 | 1,825,146 | 1,251,700 | 249,258 | 188,133 | 116,457 | 2,567,929 |
|  | [5, 50] | 5,646,789 | 5,518,738 | 5,141,588 | 857,088 | 832,699 | 690,649 | 6,503,877 |
|  | All | 43,805,192 | 11,136,559 | 7,438,239 | 3,304,273 | 1,301,558 | 881,302 | 47,109,465 |
| BBJ1k | [0, 0.05) | 33,475,711 | 5,351,085 | 959,548 | 1,601,355 | 243,654 | 47,694 | 35,077,066 |
|  | [0.05, 0.5) | 13,816,241 | 10,986,526 | 3,330,407 | 758,207 | 592,295 | 178,565 | 14,574,448 |
|  | [0.5, 1) | 1,497,074 | 1,303,683 | 801,332 | 112,479 | 95,378 | 55,523 | 1,609,553 |
|  | [1, 5) | 2,253,326 | 2,059,123 | 1,691,900 | 182,249 | 162,828 | 129,736 | 2,435,575 |
|  | [5, 50] | 5,258,811 | 5,212,583 | 5,085,955 | 431,617 | 425,821 | 402,839 | 5,690,428 |
|  | All | 56,301,163 | 24,913,000 | 11,869,142 | 3,085,907 | 1,519,976 | 814,357 | 59,387,070 |
| JEWEL3k | [0, 0.05) | 44,913,596 | 14,103,823 | 3,481,903 | 2,111,008 | 680,368 | 181,987 | 47,024,604 |
|  | [0.05, 0.5) | 11,895,969 | 10,686,265 | 4,654,377 | 661,730 | 595,772 | 276,365 | 12,557,699 |
|  | [0.5, 1) | 1,374,793 | 1,206,608 | 866,849 | 95,832 | 81,573 | 58,256 | 1,470,625 |
|  | [1, 5) | 2,194,888 | 2,041,329 | 1,775,366 | 162,166 | 148,384 | 127,112 | 2,357,054 |
|  | [5, 50] | 5,279,010 | 5,224,012 | 5,111,624 | 387,791 | 383,145 | 373,344 | 5,666,801 |
|  | All | 65,658,256 | 33,262,037 | 15,890,119 | 3,418,527 | 1,889,242 | 1,017,064 | 69,076,783 |

SNV: single nucleotide variant. Indel: short insertions and deletions. Minor allele frequency (MAF) is determined by each imputed dataset.

**Supplementary Table 2. The mean of Rsq - dosage r^2^ and the 95% confidence interval (CI) of the TOPMed, 1KGP, BBJ1k, and JEWEL3k imputation results, stratified by dosage r^2^ bins.**

|  | TOPMed | | | 1KGP | | | BBJ1k | | | JEWEL3k | | | Ratio between the mean | | |
| --- | --- | --- | --- | --- | --- | --- | --- | --- | --- | --- | --- | --- | --- | --- | --- |
| Dosage r^2^ interval | Number of variants | Mean of Rsq - dosage r^2^ | 95% CI | Number of variants | Mean of Rsq - dosage r^2^ | 95% CI | Number of variants | Mean of Rsq - dosage r^2^ | 95% CI | Number of variants | Mean of Rsq - dosage r^2^ | 95% CI | TOPMed/1KGP | TOPMed/BBJ1k | TOPMed/JEWEL3k |
| [0.5, 0.55) | 3,372 | 0.193 | (0.187, 0.200) | 3,058 | 0.024 | (0.017, 0.031) | 1,896 | 0.085 | (0.080, 0.090) | 989 | 0.105 | (0.105, 0.105) | 8.162 | 2.284 | 1.832 |
| [0.55, 0.6) | 3,543 | 0.156 | (0.150, 0.162) | 3,435 | 0.006 | (-0.000, 0.014) | 2,357 | 0.057 | (0.053, 0.063) | 1,390 | 0.070 | (0.066, 0.074) | 26.167 | 2.728 | 2.229 |
| [0.6, 0.65) | 3,992 | 0.127 | (0.120, 0.132) | 3,679 | -0.019 | (-0.029, -0.013) | 2,979 | 0.030 | (0.025, 0.035) | 1,887 | 0.043 | (0.038, 0.048) | * | 4.174 | 2.949 |
| [0.65, 0.7) | 4,265 | 0.101 | (0.095, 0.108) | 3,872 | -0.032 | (-0.041, -0.027) | 3,701 | 0.005 | (-0.001, 0.010) | 2,876 | 0.023 | (0.017, 0.028) | * | 20.684 | 4.483 |
| [0.7, 0.75) | 4,826 | 0.068 | (0.063, 0.075) | 4,322 | -0.059 | (-0.067, -0.052) | 4,575 | -0.005 | (-0.011, -0.000) | 3,927 | -0.006 | (-0.011, -0.000) | * | * | * |
| [0.75, 0.8) | 5,348 | 0.038 | (0.031, 0.043) | 5,211 | -0.069 | (-0.075, -0.061) | 5,682 | -0.021 | (-0.026, -0.015) | 4,971 | -0.014 | (-0.020, -0.010) | * | * | * |
| [0.8, 0.85) | 6,736 | 0.010 | (0.004, 0.016) | 6,369 | -0.085 | (-0.092, -0.078) | 6,989 | -0.034 | (-0.038, -0.029) | 6,792 | -0.030 | (-0.035, -0.026) | * | * | * |
| [0.85, 0.9) | 8,477 | -0.018 | (-0.023, -0.012) | 8,277 | -0.092 | (-0.099, -0.085) | 9,326 | -0.043 | (-0.048, -0.039) | 9,412 | -0.039 | (-0.044, -0.035) | * | * | * |
| [0.9, 0.95) | 13,824 | -0.042 | (-0.047, -0.037) | 13,471 | -0.089 | (-0.097, -0.084) | 14,508 | -0.051 | (-0.057, -0.047) | 14,850 | -0.045 | (-0.050, -0.041) | * | * | * |
| [0.95, 1] | 81,257 | -0.036 | (-0.040, -0.033) | 80,427 | -0.052 | (-0.057, -0.048) | 96,122 | -0.031 | (-0.034, -0.028) | 103,789 | -0.029 | (-0.032, -0.026) | * | * | * |

CI is constructed by bootstrapping variants in each dosage r^2^ bin 1,000 times. The overlapping variants imputed by the four panels are used. Dosage r^2^ is determined by each imputation result. Number of variants: the number of variants in each bin. (*) denotes not available.

**Supplementary Table 3. Metric values of rs1047781, rs113230003, and rs76246107 in the TOPMed and JEWEL3k imputation results.**

| Variant | Gene | TOPMed | | | | JEWEL3k | | | | Associated traits | PMID |
| --- | --- | --- | --- | --- | --- | --- | --- | --- | --- | --- | --- |
|  |  | Rsq | MARE | β_imp_ | Dosage r^2^ | Rsq | MARE | β_imp_ | Dosage r^2^ |  |  |
| rs1047781 (19:49206631:A:T) | *FUT2* | 0.853 | 0.196 | 0.810 | 0.774 | 0.827 | 0.091 | 0.856 | 0.89 | Psoriasis | 25574825 |
|  |  |  |  |  |  |  |  |  |  | Elevated serum carcinoembryonic antigen levels | 24941225 |
|  |  |  |  |  |  |  |  |  |  | Tumor biomarkers | 23300138 |
|  |  |  |  |  |  |  |  |  |  | Vitamin B12 levels | 22367966 |
| rs113230003 (19:18460956:G:A) | *PGPEP1* | 0.883 | 0.140 | 0.862 | 0.847 | 0.925 | 0.048 | 0.934 | 0.95 | Body mass index | 31669095 |
|  |  |  |  |  |  |  |  |  |  | Smoking initiation (ever regular vs. never regular) | 30643251 |
|  |  |  |  |  |  |  |  |  |  | Cardiovascular disease | 30595370 |
|  |  |  |  |  |  |  |  |  |  | Body mass index | 29691431 |
|  |  |  |  |  |  |  |  |  |  | Hand grip strength | 29212778 |
|  |  |  |  |  |  |  |  |  |  | Coronary artery disease | 29212778 |
| rs76246107 (19:50121274:G:A) | *PRR12* | 0.681 | 0.583 | 0.315 | 0.531 | 0.730 | 0.139 | 0.757 | 0.807 | HDL cholesterol levels | 32203549 |
|  |  |  |  |  |  |  |  |  |  | Systemic seropositive rheumatic diseases (Systemic sclerosis, systemic lupus erythematosus, rheumatoid arthritis, or idiopathic inflammatory myopathies) | 30573655 |
|  |  |  |  |  |  |  |  |  |  | Educational attainment (years of education) | 30038396 |

**Supplementary Table 4. The imputed-genotype certainty and the 95% confidence interval (CI) of the TOPMed and JEWEL3k imputation results, stratified by dosage r^2^ bins and Rsq – dosage r^2^ groups in the TOPMed imputation result.**

| Dosage r^2^ interval in the TOPMed imputation result | Rsq - dosage r^2^ in the TOPMed imputation result | Number of variants | Mean Rsq - dosage r^2^ (TOPMed) | 95% CI of mean Rsq - dosage r^2^ (TOPMed) | Mean Rsq - dosage r^2^ (JEWEL3k) | 95% CI of mean Rsq - dosage r^2^ (JEWEL3k) | Mean number of GP > 0.9 genotypes per variant (TOPMed) | 95% CI of GP > 0.9 genotypes (TOPMed) | Mean number of GP > 0.9 genotypes per variant (JEWEL3k) | 95% CI of GP > 0.9 genotypes (JEWEL3k) | Ratio of the GP > 0.9 genotypes between TOPMed and JEWEL3k |
| --- | --- | --- | --- | --- | --- | --- | --- | --- | --- | --- | --- |
| [0.5, 0.55) | [-1, -0.1) | 17 | -0.201 | (-0.258, -0.149) | -0.077 | (-0.134, -0.022) | 678.412 | (533.882, 827.588) | 901.471 | (839.000, 954.706) | 0.753 |
|  | [-0.1, 0) | 54 | -0.035 | (-0.042, -0.027) | -0.080 | (-0.104, -0.057) | 597.704 | (522.722, 684.796) | 807.593 | (753.963, 855.778) | 0.740 |
|  | [0, 0.1) | 361 | 0.062 | (0.059, 0.064) | -0.026 | (-0.036, -0.016) | 608.798 | (576.964, 637.767) | 761.161 | (740.864, 780.889) | 0.800 |
|  | [0.1, 1] | 2,314 | 0.229 | (0.226, 0.232) | -0.005 | (-0.008, -0.002) | 860.601 | (853.440, 867.914) | 893.328 | (887.741, 898.899) | 0.963 |
| [0.55, 0.6) | [-1, -0.1) | 41 | -0.181 | (-0.210, -0.155) | -0.079 | (-0.121, -0.035) | 754.244 | (657.683, 836.098) | 920.146 | (881.659, 954.268) | 0.820 |
|  | [-0.1, 0) | 117 | -0.042 | (-0.047, -0.037) | -0.060 | (-0.070, -0.050) | 557.692 | (498.872, 610.393) | 802.590 | (769.709, 833.111) | 0.695 |
|  | [0, 0.1) | 525 | 0.060 | (0.058, 0.063) | -0.033 | (-0.039, -0.027) | 661.815 | (639.303, 683.440) | 794.537 | (777.697, 810.575) | 0.833 |
|  | [0.1, 1] | 2,234 | 0.206 | (0.203, 0.209) | -0.008 | (-0.012, -0.005) | 878.802 | (872.314, 885.254) | 907.349 | (902.678, 911.870) | 0.969 |
| [0.6, 0.65) | [-1, -0.1) | 78 | -0.177 | (-0.192, -0.163) | -0.205 | (-0.235, -0.174) | 645.103 | (587.282, 701.628) | 889.564 | (861.269, 913.308) | 0.725 |
|  | [-0.1, 0) | 197 | -0.035 | (-0.039, -0.031) | -0.049 | (-0.056, -0.042) | 619.213 | (576.061, 657.528) | 808.162 | (781.838, 834.467) | 0.766 |
|  | [0, 0.1) | 864 | 0.058 | (0.057, 0.060) | -0.034 | (-0.038, -0.030) | 681.581 | (667.041, 696.958) | 802.641 | (791.519, 813.786) | 0.849 |
|  | [0.1, 1] | 2,239 | 0.187 | (0.184, 0.189) | -0.010 | (-0.013, -0.007) | 898.351 | (892.761, 903.508) | 918.086 | (913.829, 922.687) | 0.979 |
| [0.65, 0.7) | [-1, -0.1) | 103 | -0.191 | (-0.208, -0.176) | -0.146 | (-0.166, -0.126) | 577.243 | (516.757, 637.835) | 839.495 | (808.786, 870.660) | 0.688 |
|  | [-0.1, 0) | 261 | -0.038 | (-0.042, -0.035) | -0.049 | (-0.056, -0.042) | 620.862 | (587.759, 657.471) | 806.912 | (787.172, 828.100) | 0.769 |
|  | [0, 0.1) | 1,105 | 0.059 | (0.057, 0.061) | -0.028 | (-0.031, -0.024) | 750.582 | (738.810, 762.243) | 853.919 | (846.039, 860.941) | 0.879 |
|  | [0.1, 1] | 2,154 | 0.165 | (0.164, 0.167) | -0.012 | (-0.015, -0.009) | 905.473 | (900.660, 910.046) | 926.041 | (922.439, 929.936) | 0.978 |
| [0.7, 0.75) | [-1, -0.1) | 172 | -0.203 | (-0.217, -0.190) | -0.161 | (-0.181, -0.142) | 614.640 | (571.326, 662.558) | 855.279 | (830.076, 879.174) | 0.719 |
|  | [-0.1, 0) | 470 | -0.036 | (-0.039, -0.034) | -0.042 | (-0.048, -0.037) | 683.409 | (662.068, 703.785) | 847.994 | (835.602, 859.711) | 0.806 |
|  | [0, 0.1) | 1,771 | 0.058 | (0.056, 0.059) | -0.026 | (-0.029, -0.024) | 790.372 | (782.839, 797.941) | 873.635 | (868.814, 878.800) | 0.905 |
|  | [0.1, 1] | 1,804 | 0.150 | (0.148, 0.151) | -0.014 | (-0.017, -0.010) | 938.183 | (934.830, 941.696) | 947.578 | (944.943, 950.366) | 0.990 |
| [0.75, 0.8) | [-1, -0.1) | 296 | -0.196 | (-0.208, -0.184) | -0.124 | (-0.138, -0.112) | 590.777 | (559.088, 625.368) | 845.554 | (824.861, 864.341) | 0.699 |
|  | [-0.1, 0) | 795 | -0.039 | (-0.041, -0.037) | -0.040 | (-0.043, -0.037) | 726.355 | (712.467, 739.679) | 875.086 | (867.709, 883.096) | 0.830 |
|  | [0, 0.1) | 2,433 | 0.052 | (0.051, 0.053) | -0.025 | (-0.027, -0.023) | 832.725 | (827.040, 837.900) | 896.147 | (892.358, 899.942) | 0.929 |
|  | [0.1, 1] | 1,219 | 0.135 | (0.133, 0.136) | -0.011 | (-0.014, -0.007) | 961.262 | (958.590, 963.821) | 964.872 | (962.858, 966.940) | 0.996 |
| [0.8, 0.85) | [-1, -0.1) | 444 | -0.198 | (-0.206, -0.190) | -0.118 | (-0.127, -0.110) | 637.750 | (612.203, 661.257) | 877.696 | (865.957, 889.572) | 0.727 |
|  | [-0.1, 0) | 1,534 | -0.038 | (-0.039, -0.036) | -0.044 | (-0.046, -0.042) | 762.407 | (753.995, 770.950) | 881.831 | (875.794, 887.501) | 0.865 |
|  | [0, 0.1) | 3,437 | 0.046 | (0.045, 0.047) | -0.020 | (-0.022, -0.019) | 878.940 | (875.420, 882.404) | 923.727 | (921.254, 926.233) | 0.952 |
|  | [0.1, 1] | 648 | 0.123 | (0.122, 0.124) | -0.009 | (-0.013, -0.006) | 979.775 | (978.125, 981.344) | 978.927 | (977.625, 980.235) | 1.001 |
| [0.85, 0.9) | [-1, -0.1) | 708 | -0.197 | (-0.204, -0.190) | -0.113 | (-0.119, -0.106) | 639.355 | (621.524, 656.051) | 878.624 | (869.178, 888.466) | 0.728 |
|  | [-0.1, 0) | 3,068 | -0.034 | (-0.035, -0.033) | -0.038 | (-0.040, -0.037) | 815.883 | (811.860, 820.626) | 902.096 | (898.836, 905.459) | 0.904 |
|  | [0, 0.1) | 4,002 | 0.035 | (0.035, 0.036) | -0.016 | (-0.017, -0.015) | 917.337 | (915.179, 919.517) | 946.765 | (944.889, 948.524) | 0.969 |
|  | [0.1, 1] | 90 | 0.110 | (0.108, 0.112) | 0.003 | (-0.008, 0.014) | 988.122 | (986.500, 989.511) | 984.856 | (981.833, 987.033) | 1.003 |
| [0.9, 0.95) | [-1, -0.1) | 1,605 | -0.197 | (-0.202, -0.191) | -0.112 | (-0.117, -0.108) | 722.164 | (712.504, 731.718) | 915.759 | (910.085, 921.125) | 0.789 |
|  | [-0.1, 0) | 7,369 | -0.036 | (-0.036, -0.035) | -0.030 | (-0.031, -0.030) | 875.934 | (874.080, 877.752) | 937.342 | (935.741, 939.160) | 0.934 |
|  | [0, 0.1] | 4,257 | 0.022 | (0.022, 0.023) | -0.010 | (-0.011, -0.009) | 951.852 | (950.696, 952.971) | 968.846 | (968.034, 969.702) | 0.982 |
| [0.95, 1] | [-1, -0.1) | 4,698 | -0.171 | (-0.173, -0.169) | -0.105 | (-0.107, -0.102) | 810.871 | (806.938, 814.650) | 954.243 | (951.860, 956.460) | 0.850 |
|  | [-0.1, 0) | 66,274 | -0.028 | (-0.028, -0.027) | -0.017 | (-0.017, -0.017) | 960.951 | (960.628, 961.276) | 982.272 | (982.083, 982.464) | 0.978 |
|  | [0, 0.05] | 6,325 | 0.007 | (0.007, 0.007) | -0.001 | (-0.001, -0.000) | 984.671 | (984.383, 984.962) | 988.399 | (988.179, 988.615) | 0.996 |

CI is constructed by bootstrapping variants in each group 1,000 times. Number of variants: the number of variants in each bin. Genotype certainty is determined by the imputed genotype probability (GP).

**Supplementary Table 5. Metric values of rs1047781, rs113230003, and rs76246107 using the 1KGP reference panel with seven scalings of the θ value.**

| Variant | Gene | Scaling of the θ value | Rsq | MARE | β_imp_ | Dosage r^2^ |
| --- | --- | --- | --- | --- | --- | --- |
| rs1047781 (19:49206631:A:T) | *FUT2* | 0.01 | 0.789 | 0.297 | 0.715 | 0.629 |
|  |  | 0.1 | 0.754 | 0.253 | 0.717 | 0.668 |
|  |  | 0.5 | 0.700 | 0.203 | 0.705 | 0.71 |
|  |  | 1 | 0.654 | 0.179 | 0.683 | 0.725 |
|  |  | 2 | 0.564 | 0.151 | 0.625 | 0.73 |
|  |  | 10 | 0.011 | 0.007 | 0.042 | 0.313 |
|  |  | 100 | 0.001 | 0.001 | -0.001 | 0.002 |
| rs113230003 (19:18460956:G:A) | *PGPEP1* | 0.01 | 0.932 | 0.163 | 0.888 | 0.833 |
|  |  | 0.1 | 0.923 | 0.148 | 0.892 | 0.847 |
|  |  | 0.5 | 0.907 | 0.137 | 0.890 | 0.856 |
|  |  | 1 | 0.893 | 0.130 | 0.888 | 0.862 |
|  |  | 2 | 0.876 | 0.129 | 0.879 | 0.86 |
|  |  | 10 | 0.700 | 0.151 | 0.746 | 0.793 |
|  |  | 100 | 0.112 | 0.043 | 0.262 | 0.631 |
| rs76246107 (19:50121274:G:A) | *PRR12* | 0.01 | 0.732 | 0.365 | 0.580 | 0.484 |
|  |  | 0.1 | 0.673 | 0.316 | 0.566 | 0.509 |
|  |  | 0.5 | 0.566 | 0.249 | 0.527 | 0.536 |
|  |  | 1 | 0.478 | 0.206 | 0.483 | 0.545 |
|  |  | 2 | 0.343 | 0.156 | 0.388 | 0.518 |
|  |  | 10 | 0.059 | 0.038 | 0.109 | 0.314 |
|  |  | 100 | 0.052 | 0.039 | 0.079 | 0.196 |

**Supplementary Table 6. The mean of Rsq, EmpRsq, and Rsq – EmpRsq, and the number of confident alleles, and the 95% confidence interval (CI) for array markers and imputed variants.**

|  | Markers on the genotyping array (N = 10,273) | | | | | | | | All imputed varaints (N = 1,084,535) | | | |
| --- | --- | --- | --- | --- | --- | --- | --- | --- | --- | --- | --- | --- |
| The θ scaling | Mean Rsq | 95% CI | Mean EmpRsq | 95% CI | Mean of Rsq - EmpRsq | 95% CI | Total Matches with HDS > 0.9 | 95% CI | Mean Rsq | 95% CI | Total Matches with HDS > 0.9 | 95% CI |
| 0.01 | 0.924 | (0.922, 0.926) | 0.861 | (0.857, 0.865) | 0.063 | (0.061, 0.065) | 7,038,498 | (6,934,233, 7,146,437) | 0.281 | (0.281, 0.282) | 111,900,629 | (111,238,044, 112,579,382) |
| 0.02 | 0.920 | (0.918, 0.922) | 0.864 | (0.860, 0.868) | 0.056 | (0.054, 0.058) | 7,017,820 | (6,911,633, 7,121,419) | 0.275 | (0.274, 0.276) | 111,419,293 | (110,780,812, 112,032,297) |
| 0.1 | 0.906 | (0.904, 0.909) | 0.871 | (0.867, 0.875) | 0.035 | (0.033, 0.037) | 6,945,870 | (6,835,512, 7,048,447) | 0.255 | (0.254, 0.256) | 109,378,239 | (108,740,965, 110,012,865) |
| 0.125 | 0.904 | (0.901, 0.906) | 0.872 | (0.868, 0.876) | 0.032 | (0.030, 0.033) | 6,932,419 | (6,825,416, 7,033,614) | 0.251 | (0.251, 0.252) | 108,895,828 | (108,250,918, 109,567,220) |
| 0.2 | 0.897 | (0.894, 0.900) | 0.874 | (0.870, 0.878) | 0.023 | (0.021, 0.025) | 6,898,693 | (6,789,937, 7,001,984) | 0.243 | (0.242, 0.244) | 107,693,098 | (107,042,078, 108,305,147) |
| 0.25 | 0.893 | (0.890, 0.896) | 0.875 | (0.871, 0.879) | 0.018 | (0.016, 0.020) | 6,878,411 | (6,781,058, 6,968,745) | 0.238 | (0.238, 0.239) | 107,009,589 | (106,357,639, 107,658,135) |
| 0.33 | 0.887 | (0.885, 0.890) | 0.876 | (0.872, 0.880) | 0.011 | (0.010, 0.013) | 6,850,978 | (6,753,853, 6,958,903) | 0.232 | (0.231, 0.233) | 106,046,221 | (105,387,228, 106,652,887) |
| 0.5 | 0.877 | (0.874, 0.880) | 0.877 | (0.874, 0.881) | -0.001 | (-0.002, 0.001) | 6,798,453 | (6,692,889, 6,907,488) | 0.222 | (0.221, 0.222) | 104,270,414 | (103,673,654, 104,896,822) |
| 0.67 | 0.867 | (0.864, 0.870) | 0.878 | (0.874, 0.882) | -0.011 | (-0.013, -0.009) | 6,750,338 | (6,638,260, 6,859,075) | 0.213 | (0.213, 0.214) | 102,479,229 | (101,882,033, 103,093,994) |
| 0.8 | 0.860 | (0.856, 0.863) | 0.878 | (0.874, 0.882) | -0.018 | (-0.020, -0.017) | 6,713,647 | (6,610,496, 6,818,303) | 0.208 | (0.207, 0.208) | 101,026,131 | (100,417,746, 101,605,202) |
| 1 | 0.849 | (0.845, 0.852) | 0.878 | (0.874, 0.882) | -0.029 | (-0.031, -0.027) | 6,656,909 | (6,540,177, 6,764,048) | 0.200 | (0.200, 0.201) | 98,875,870 | (98,310,932, 99,512,240) |
| 1.25 | 0.835 | (0.831, 0.839) | 0.877 | (0.873, 0.881) | -0.042 | (-0.044, -0.040) | 6,583,089 | (6,485,653, 6,685,346) | 0.192 | (0.192, 0.193) | 95,910,630 | (95,280,692, 96,484,670) |
| 1.5 | 0.821 | (0.817, 0.825) | 0.876 | (0.871, 0.880) | -0.055 | (-0.056, -0.053) | 6,499,920 | (6,384,959, 6,612,093) | 0.185 | (0.184, 0.185) | 92,957,791 | (92,371,537, 93,558,904) |
| 2 | 0.792 | (0.788, 0.796) | 0.871 | (0.867, 0.875) | -0.079 | (-0.082, -0.077) | 6,329,549 | (6,222,556, 6,438,919) | 0.172 | (0.171, 0.172) | 87,221,001 | (86,660,214, 87,783,280) |
| 3 | 0.733 | (0.728, 0.737) | 0.860 | (0.856, 0.864) | -0.127 | (-0.130, -0.125) | 5,934,895 | (5,824,701, 6,049,354) | 0.150 | (0.150, 0.151) | 75,388,680 | (74,866,458, 75,934,704) |
| 4 | 0.674 | (0.668, 0.679) | 0.845 | (0.841, 0.849) | -0.171 | (-0.174, -0.168) | 5,499,890 | (5,392,888, 5,617,340) | 0.133 | (0.133, 0.134) | 64,177,890 | (63,743,167, 64,664,822) |
| 5 | 0.620 | (0.614, 0.626) | 0.829 | (0.824, 0.834) | -0.209 | (-0.212, -0.206) | 5,073,507 | (4,967,022, 5,178,694) | 0.120 | (0.119, 0.120) | 54,572,785 | (54,125,554, 54,993,528) |
| 8 | 0.499 | (0.493, 0.505) | 0.786 | (0.781, 0.792) | -0.287 | (-0.291, -0.283) | 4,095,593 | (3,991,923, 4,203,283) | 0.097 | (0.097, 0.098) | 38,237,092 | (37,905,042, 38,608,968) |
| 10 | 0.442 | (0.436, 0.449) | 0.763 | (0.758, 0.769) | -0.320 | (-0.325, -0.316) | 3,635,130 | (3,530,229, 3,734,559) | 0.089 | (0.088, 0.089) | 32,070,626 | (31,744,424, 32,410,211) |
| 50 | 0.145 | (0.140, 0.149) | 0.537 | (0.530, 0.544) | -0.392 | (-0.398, -0.386) | 1,305,905 | (1,228,055, 1,379,842) | 0.056 | (0.056, 0.057) | 17,265,686 | (17,013,172, 17,511,169) |
| 100 | 0.083 | (0.080, 0.087) | 0.426 | (0.419, 0.433) | -0.343 | (-0.349, -0.336) | 859,124 | (799,101, 924,626) | 0.052 | (0.052, 0.052) | 16,297,937 | (16,054,772, 16,540,828) |

CI is constructed by bootstrapping variants in each group 1,000 times. Confident allele is defined as an allele imputed with haploid dosage (HDS) > 0.9.

**Supplementary Table 7. Number of confident alleles and high-Rsq variants using the 1KGP reference panel and 21 scalings of the θ value.**

| Scaling of θ value | Alleles with HDS > 0.9 | | | Variants with Rsq > 0.7 | | |
| --- | --- | --- | --- | --- | --- | --- |
|  | All | EAS-only | Non-EAS | All | EAS-only | Non-EAS |
| 0.01 | 111,900,629 | 353,418 | 41,140 | 214,832 | 20,923 | 7,213 |
| 0.02 | 111,419,293 | 340,835 | 34,699 | 209,018 | 19,440 | 5,858 |
| 0.1 | 109,378,239 | 303,045 | 19,783 | 194,113 | 15,882 | 2,999 |
| 0.125 | 108,895,828 | 295,656 | 18,003 | 191,782 | 15,301 | 2,665 |
| 0.2 | 107,693,098 | 278,442 | 14,059 | 186,726 | 14,019 | 2,072 |
| 0.25 | 107,009,589 | 268,947 | 12,108 | 184,184 | 13,428 | 1,864 |
| 0.33 | 106,046,221 | 255,937 | 9,914 | 180,399 | 12,606 | 1,569 |
| 0.5 | 104,270,414 | 233,269 | 7,111 | 173,640 | 11,198 | 1,218 |
| 0.67 | 102,479,229 | 215,508 | 5,251 | 168,236 | 10,220 | 936 |
| 0.8 | 101,026,131 | 203,389 | 4,356 | 164,512 | 9,544 | 819 |
| 1 | 98,875,870 | 185,870 | 3,362 | 159,469 | 8,627 | 724 |
| 1.25 | 95,910,630 | 166,420 | 2,636 | 153,657 | 7,626 | 608 |
| 1.5 | 92,957,791 | 148,619 | 2,031 | 147,868 | 6,821 | 490 |
| 2 | 87,221,001 | 118,354 | 1,325 | 137,454 | 5,400 | 371 |
| 3 | 75,388,680 | 70,260 | 785 | 118,479 | 3,210 | 264 |
| 4 | 64,177,890 | 38,507 | 662 | 101,666 | 1,826 | 203 |
| 5 | 54,572,785 | 22,061 | 567 | 88,379 | 1,118 | 175 |
| 8 | 38,237,092 | 10,462 | 445 | 65,357 | 594 | 147 |
| 10 | 32,070,626 | 9,027 | 469 | 56,827 | 525 | 140 |
| 50 | 17,265,686 | 7,431 | 456 | 30,874 | 391 | 119 |
| 100 | 16,297,937 | 7,423 | 443 | 28,538 | 387 | 117 |

The number of EAS-only and non-EAS variants were 83,023 and 696,396.

**Supplementary Table 8. Number of variants, confident alleles, and high-Rsq variants using using the simulated EUR-EAS reference panels with different sizes and EUR proportions, and 100 EUR as the target sample.**

| Reference panel | Number of variants in reference panel | | Alleles with HDS > 0.9 | | | Variants with Rsq > 0.7 | | |
| --- | --- | --- | --- | --- | --- | --- | --- | --- |
|  | All | Non-EUR | All | EUR-only | Non-EUR | All | EUR-only | Non-EUR |
| 1KGP-EURn403 | 456,835 | * | 8,670,087 | 84,797 | * | 163,468 | 18,646 | * |
| 1KGP-EURn403+1KGP-EAS | 815,407 | 358,572 | 8,523,623 | 85,099 | 181 | 168,046 | 20,811 | 242 |
| 1KGP-EURn403+1KGP-EAS+500JPT | 912,663 | 455,828 | 8,697,251 | 90,731 | 260 | 179,340 | 23,221 | 296 |
| 1KGP-EURn403+1KGP-EAS+1000JPT | 978,746 | 521,911 | 8,787,978 | 93,271 | 284 | 184,613 | 24,472 | 308 |
| 1KGP-EURn403+1KGP-EAS+1500JPT | 1,030,657 | 573,822 | 8,901,041 | 96,251 | 337 | 190,618 | 25,731 | 395 |
| 1KGP-EURn403+1KGP-EAS+2000JPT | 1,073,079 | 616,244 | 8,930,669 | 97,674 | 363 | 193,091 | 26,392 | 432 |
| 1KGP-EURn403+1KGP-EAS+2500JPT | 1,108,702 | 651,867 | 8,969,106 | 99,053 | 405 | 195,843 | 27,024 | 458 |
| 1KGP-EURn403+1KGP-EAS+3256JPT | 1,152,852 | 696,017 | 9,039,186 | 101,116 | 458 | 200,161 | 27,941 | 521 |

The number of EUR-only variants was 114,606. (*) denotes not available. EURn403 represents the 403 EUR; 1KGP-EAS represents the 504 EAS; 500–3256JPT represents the number of JPT samples in the reference panel.

**Supplementary Table 9. Number of variants, confident alleles, and high-Rsq variants using the simulated JPT-1KGP reference panels with different sizes and ancestral diversities, and WGS_993_ as the target sample.**

| Reference panel | Number of variants in reference panel | | Alleles with HDS > 0.9 | | | | Variants with Rsq > 0.7 | | | |
| --- | --- | --- | --- | --- | --- | --- | --- | --- | --- | --- |
|  | All | Non-EAS | All | JPT_3256_-only | 1KGP-EAS-only | Non-EAS | All | JPT_3256_-only | 1KGP-EAS-only | Non-EAS |
| 100JPT | 295,966 | * | * | * | * | * | 136,682 | * | * | * |
| 500JPT | 510,113 | * | * | * | * | * | 178,534 | * | * | * |
| 1000JPT | 662,071 | * | * | * | * | * | 210,849 | * | * | * |
| 1500JPT | 769,906 | * | * | * | * | * | 232,582 | * | * | * |
| 2000JPT | 855,094 | * | * | * | * | * | 249,553 | * | * | * |
| 2500JPT | 924,533 | * | * | * | * | * | 261,409 | * | * | * |
| 3256JPT | 1,010,230 | * | 96,451,515 | 24,599 | * | * | 274,343 | 10,613 | * | * |
| 3256JPT+1KGP-JPT | 1,011,682 | * | 96,286,919 | 24,171 | * | * | 272,521 | 10,554 | * | * |
| 3256JPT+1KGP-EAS | 1,038,246 | * | 96,190,813 | 24,151 | 159 | * | 271,937 | 10,884 | 158 | * |
| 3256JPT+1KGP-EAS+EUR | 1,163,109 | 124,863 | 95,590,037 | 23,488 | 138 | 183 | 266,300 | 10,692 | 144 | 184 |
| 3256JPT+1KGP-EAS+EUR+AFR | 1,471,007 | 432,761 | 95,062,865 | 23,891 | 143 | 200 | 267,008 | 10,893 | 143 | 219 |
| 3256JPT+1KGP-EAS+EUR+AFR+AMR | 1,486,412 | 448,166 | 94,503,390 | 23,383 | 130 | 229 | 263,233 | 10,775 | 137 | 201 |
| 3256JPT+1KGP-All | 1,533,735 | 495,489 | 94,488,850 | 23,666 | 131 | 254 | 264,221 | 11,015 | 142 | 265 |

The number of JPT_3256_-only and 1KGP-EAS-only variants was 74,490 and 7,627. (*) denotes not available. 100–3256 JPT represents the number of JPT samples in the reference panel; 1KGP- followed by the ancestry represents the 1KGP subset.

**Supplementary Table 10. The total θ value estimated by Minimac3 or transformed from the HapMap2 genetic map using Minimac4 v1.0.2.**

| Reference panel | Parameter source | Background template switching rate | Number of variants with a background template switching rate | Number of variants not with the background template switching rate | Total θ value | Ratio between the transformed and estimated total θ values |
| --- | --- | --- | --- | --- | --- | --- |
| 1KGP | Genetic map and Minimac4 | 1.00E-05 | 493,335 | 591,200 | 110.4 | 0.152 |
| JEWEL3k | Genetic map and Minimac4 | 1.00E-05 | 792,501 | 741,234 | 112.6 | 0.267 |
| 1KGP | Minimac3 | 0.00065347 | 1,084,368 | 166 | 728 | * |
| JEWEL3k | Minimac3 | 0.00027504 | 1,533,730 | 4 | 422.4 | * |

Background template switching rate is a minimum probability of switching Minimac sets for all markers. (*) denotes not available.

**Supplementary Table 11. The mean EmpRsq and Rsq using 1KGP panel with 21 scalings of the θ value (chr20).**

| Scaling of θ value | MAF ≥ 5% | | 5% > MAF ≥ 1% | | 1% > MAF ≥ 0.5% | |
| --- | --- | --- | --- | --- | --- | --- |
|  | EmpRsq | Rsq | EmpRsq | Rsq | EmpRsq | Rsq |
| 0.01 | 0.896 | 0.946 | 0.842 | 0.906 | 0.767 | 0.855 |
| 0.02 | 0.899 | 0.944 | 0.845 | 0.902 | 0.772 | 0.849 |
| 0.1 | 0.904 | 0.936 | 0.854 | 0.886 | 0.781 | 0.826 |
| 0.125 | 0.905 | 0.934 | 0.855 | 0.883 | 0.784 | 0.822 |
| 0.2 | 0.906 | 0.930 | 0.857 | 0.876 | 0.786 | 0.809 |
| 0.25 | 0.907 | 0.928 | 0.858 | 0.871 | 0.788 | 0.802 |
| 0.33 | 0.908 | 0.925 | 0.858 | 0.865 | 0.789 | 0.793 |
| 0.5 | 0.909 | 0.920 | 0.859 | 0.854 | 0.791 | 0.772 |
| 0.67 | 0.910 | 0.915 | 0.860 | 0.843 | 0.792 | 0.757 |
| 0.8 | 0.911 | 0.911 | 0.859 | 0.836 | 0.795 | 0.747 |
| 1 | 0.911 | 0.906 | 0.859 | 0.825 | 0.795 | 0.730 |
| 1.25 | 0.911 | 0.899 | 0.858 | 0.812 | 0.794 | 0.712 |
| 1.5 | 0.911 | 0.893 | 0.857 | 0.799 | 0.795 | 0.694 |
| 2 | 0.911 | 0.880 | 0.853 | 0.773 | 0.790 | 0.659 |
| 3 | 0.909 | 0.853 | 0.845 | 0.723 | 0.774 | 0.590 |
| 4 | 0.905 | 0.824 | 0.836 | 0.676 | 0.759 | 0.531 |
| 5 | 0.900 | 0.793 | 0.824 | 0.631 | 0.740 | 0.477 |
| 8 | 0.881 | 0.701 | 0.784 | 0.511 | 0.679 | 0.345 |
| 10 | 0.867 | 0.644 | 0.755 | 0.447 | 0.642 | 0.289 |
| 50 | 0.674 | 0.223 | 0.451 | 0.110 | 0.309 | 0.045 |
| 100 | 0.557 | 0.118 | 0.329 | 0.058 | 0.215 | 0.021 |

Minor allele frequency (MAF) is determined by the genotyping array.

**Supplementary Table 12. Number of ancestry specific variants using the 1KGP reference panel and 21 scalings of the θ value (chr20).**

| Scaling of θ value | Matches with HDS > 0.9 | | | Variants with Rsq > 0.7 | | |
| --- | --- | --- | --- | --- | --- | --- |
|  | All | EAS-only | Non-EAS | All | EAS-only | Non-EAS |
| 0.01 | 98,727,681 | 352,684 | 43,333 | 183,237 | 20,210 | 7,541 |
| 0.02 | 98,572,403 | 341,218 | 38,032 | 178,863 | 18,795 | 6,272 |
| 0.1 | 97,978,045 | 305,535 | 24,863 | 168,330 | 15,387 | 3,754 |
| 0.125 | 97,851,883 | 299,290 | 22,877 | 166,752 | 14,890 | 3,455 |
| 0.2 | 97,499,088 | 284,672 | 19,009 | 163,413 | 13,794 | 2,883 |
| 0.25 | 97,275,574 | 277,091 | 17,297 | 161,693 | 13,215 | 2,599 |
| 0.33 | 96,978,698 | 266,938 | 15,209 | 159,540 | 12,516 | 2,330 |
| 0.5 | 96,415,508 | 249,500 | 12,297 | 155,773 | 11,398 | 1,858 |
| 0.67 | 95,920,534 | 235,502 | 10,573 | 152,868 | 10,567 | 1,591 |
| 0.8 | 95,551,457 | 226,266 | 9,655 | 151,027 | 10,045 | 1,435 |
| 1 | 95,005,923 | 213,572 | 8,302 | 148,454 | 9,348 | 1,269 |
| 1.25 | 94,288,606 | 199,228 | 7,170 | 145,675 | 8,566 | 1,138 |
| 1.5 | 93,522,393 | 186,060 | 6,322 | 143,043 | 7,905 | 1,023 |
| 2 | 91,944,606 | 163,113 | 5,245 | 138,556 | 6,842 | 847 |
| 3 | 88,112,259 | 123,997 | 3,968 | 130,108 | 5,153 | 639 |
| 4 | 83,207,487 | 92,519 | 3,287 | 122,005 | 3,922 | 537 |
| 5 | 77,823,599 | 67,838 | 2,741 | 114,110 | 2,911 | 471 |
| 8 | 62,370,754 | 26,440 | 2,006 | 92,659 | 1,219 | 384 |
| 10 | 54,155,330 | 15,200 | 1,858 | 82,081 | 735 | 359 |
| 50 | 20,743,295 | 3,452 | 618 | 34,655 | 194 | 108 |
| 100 | 18,251,592 | 3,422 | 568 | 30,267 | 192 | 97 |

The number of EAS-only and non-EAS variants were 81,922 and 655,794.

**Supplementary Table 13. The θ value estimation in JPT and JPT-1KGP reference panels (chr20).**

| Subset | Size | EAS (%) | Total θ value | SD |
| --- | --- | --- | --- | --- |
| 100JPT | 100 | 100 | 712.547 | 0.000 |
| 500JPT | 500 | 100 | 444.486 | 2.945 |
| 1000JPT | 1,000 | 100 | 374.091 | 3.875 |
| 1500JPT | 1,500 | 100 | 338.074 | 2.539 |
| 2000JPT | 2,000 | 100 | 317.818 | 2.551 |
| 2500JPT | 2,500 | 100 | 303.009 | 2.074 |
| 3256JPT | 3,256 | 100 | 288.416 | 3.108 |
| 3256JPT+1KGP-JPT | 3,360 | 100 | 287.029 | 3.083 |
| 3256JPT+1KGP-EAS | 3,760 | 100 | 319.261 | 12.359 |
| 3256JPT+1KGP-EAS+EUR | 4,263 | 88.2 | 348.218 | 8.846 |
| 3256JPT+1KGP-EAS+EUR+AFR | 4,924 | 76.36 | 394.002 | 18.275 |
| 3256JPT+1KGP-EAS+EUR+AFR+AMR | 5,217 | 72.07 | 407.478 | 18.932 |
| 3256JPT+1KGP-EAS+EUR+AFR+AMR+SAS | 5,760 | 65.28 | 432.273 | 19.244 |

The total θ value and SD show the mean and sample standard deviation of 10 runs. The number followed by JPT represents the number of JPT samples in the subset of 3256 JPT WGS.

**Supplementary Table 14. The θ value estimation in downsampled 1KGP reference panels (chr20).**

| Subset | Size | EAS (%) | Total θ value | SD |
| --- | --- | --- | --- | --- |
| EAS | 504 | 100 | 878.400 | 0.894 |
| EAS+EUR | 504 | ≈50.05 | 677.187 | 11.752 |
| EAS+EUR+AFR | 504 | ≈30.22 | 768.872 | 20.964 |
| EAS+EUR+AFR+AMR | 504 | ≈25.01 | 767.134 | 12.200 |
| EAS+EUR+AFR+AMR+SAS | 504 | ≈20.13 | 795.409 | 18.184 |

The total θ value and SD show the mean and sample standard deviation of 10 runs.

**Supplementary Table 15. The θ value estimation in EUR-EAS reference panels (chr20).**

| Subset | Size | EUR (%) | Total θ value | SD |
| --- | --- | --- | --- | --- |
| 1KGP-EURn403 | 403 | 100 | 700.953 | 6.272 |
| 1KGP-EURn403+1KGP-EAS | 907 | 44.43 | 666.314 | 6.589 |
| 1KGP-EURn403+1KGP-EAS+500JPT | 1,407 | 28.64 | 550.759 | 15.821 |
| 1KGP-EURn403+1KGP-EAS+1000JPT | 1,907 | 21.13 | 472.784 | 11.923 |
| 1KGP-EURn403+1KGP-EAS+1500JPT | 2,407 | 16.74 | 432.866 | 17.586 |
| 1KGP-EURn403+1KGP-EAS+2000JPT | 2,907 | 13.86 | 405.770 | 8.779 |
| 1KGP-EURn403+1KGP-EAS+2500JPT | 3,407 | 11.83 | 374.944 | 8.765 |
| 1KGP-EURn403+1KGP-EAS+3256JPT | 4,163 | 9.68 | 351.788 | 14.172 |

The total θ value and SD show the mean and sample standard deviation of 10 runs. EURn403 represents the 403 EUR samples in 1KGP.

**Supplementary Table 16. The θ value estimation in the 1KGP reference panel subsets (chr20).**

| Subset | Size | EAS (%) | Total θ value | SD |
| --- | --- | --- | --- | --- |
| JPT | 104 | 100 | 878.400 | 0.894 |
| EAS | 504 | 100 | 689.087 | 4.364 |
| EAS+EUR | 1,007 | 50.05 | 574.468 | 10.214 |
| EAS+EUR+AFR | 1,668 | 30.22 | 553.459 | 13.163 |
| EAS+EUR+AFR+AMR | 2,015 | 25.01 | 525.222 | 8.794 |
| EAS+EUR+AFR+AMR+SAS | 2,504 | 20.13 | 518.581 | 10.804 |

The total θ value and SD show the mean and sample standard deviation of 10 runs.

**Supplementary Table 17. Reference panel description, the total θ value estimated by Minimac3, the mean EmpRsq, and the mean Rsq using the simulated EUR-EAS reference panels with different sizes and EUR proportions, and 100 EUR as the target sample (chr20).**

| Reference panel | Reference panel size | EUR (%) | Total θ value | MAF ≥ 5% | | 5% > MAF ≥ 1% | |
| --- | --- | --- | --- | --- | --- | --- | --- |
|  |  |  |  | EmpRsq | Rsq | EmpRsq | Rsq |
| 1KGP-EURn403 | 403 | 100 | 693.740 | 0.842 | 0.799 | 0.688 | 0.634 |
| 1KGP-EURn403+1KGP-EAS | 907 | 44.43 | 671.069 | 0.834 | 0.796 | 0.678 | 0.654 |
| 1KGP-EURn403+1KGP-EAS+500JPT | 1,407 | 28.64 | 545.358 | 0.829 | 0.812 | 0.671 | 0.686 |
| 1KGP-EURn403+1KGP-EAS+1000JPT | 1,907 | 21.13 | 491.028 | 0.826 | 0.818 | 0.665 | 0.698 |
| 1KGP-EURn403+1KGP-EAS+1500JPT | 2,407 | 16.74 | 427.300 | 0.823 | 0.826 | 0.662 | 0.714 |
| 1KGP-EURn403+1KGP-EAS+2000JPT | 2,907 | 13.86 | 404.376 | 0.821 | 0.829 | 0.659 | 0.721 |
| 1KGP-EURn403+1KGP-EAS+2500JPT | 3,407 | 11.83 | 374.580 | 0.819 | 0.833 | 0.658 | 0.728 |
| 1KGP-EURn403+1KGP-EAS+3256JPT | 4,163 | 9.68 | 329.281 | 0.817 | 0.838 | 0.655 | 0.737 |

EURn403 represents the 403 EUR; 1KGP-EAS represents the 504 EAS; 500–3256JPT represents the number of JPT samples in the reference panel. Minor allele frequency (MAF) is determined by the genotyping array.

**Supplementary Table 18. Number of variants using the simulated EUR-EAS reference panels with different sizes and EUR proportions, and 100 EUR as the target sample (chr20).**

| Reference panel | Number of variants in reference panel | | Matches with HDS > 0.9 | | | Variants with Rsq > 0.7 | | |
| --- | --- | --- | --- | --- | --- | --- | --- | --- |
|  | All | Non-EUR | All | EUR-only | Non-EUR | All | EUR-only | Non-EUR |
| 1KGP-EURn403 | 413,061 | * | 7,965,898 | 83,338 | * | 148,574 | 16,860 | * |
| 1KGP-EURn403+1KGP-EAS | 765,672 | 352,611 | 7,907,608 | 83,965 | 397 | 151,194 | 18,554 | 419 |
| 1KGP-EURn403+1KGP-EAS+500JPT | 864,017 | 450,956 | 8,037,885 | 89,063 | 487 | 158,587 | 20,278 | 569 |
| 1KGP-EURn403+1KGP-EAS+1000JPT | 929,555 | 516,494 | 8,078,519 | 91,544 | 539 | 162,349 | 21,185 | 578 |
| 1KGP-EURn403+1KGP-EAS+1500JPT | 980,637 | 567,576 | 8,137,404 | 93,860 | 628 | 166,193 | 22,093 | 644 |
| 1KGP-EURn403+1KGP-EAS+2000JPT | 1,021,496 | 608,435 | 8,141,740 | 94,913 | 725 | 167,766 | 22,497 | 676 |
| 1KGP-EURn403+1KGP-EAS+2500JPT | 1,056,632 | 643,571 | 8,158,317 | 96,006 | 764 | 169,656 | 22,956 | 714 |
| 1KGP-EURn403+1KGP-EAS+3256JPT | 1,100,366 | 687,305 | 8,198,561 | 97,316 | 870 | 172,228 | 23,589 | 791 |

The number of EUR-only variants was 107,380. (*) denotes not available. EURn403 represents the 403 EUR; 1KGP-EAS represents the 504 EAS; 500–3256JPT represents the number of JPT samples in the reference panel.

**Supplementary Table 19. Reference panel description, the total θ value estimated by Minimac3, the mean EmpRsq, and the mean Rsq using the simulated JPT-1KGP reference panels with different sizes and ancestral diversity, and WGS_993_ as the target sample (chr20).**

| Reference panel | Reference panel size | EAS (%) | Total θ value | MAF ≥ 5% | | 5% > MAF ≥ 1% | | 1% > MAF ≥ 0.5% | |
| --- | --- | --- | --- | --- | --- | --- | --- | --- | --- |
|  |  |  |  | EmpRsq | Rsq | EmpRsq | Rsq | EmpRsq | Rsq |
| 3256JPT | 3,256 | 100 | 292.980 | 0.968 | 0.964 | 0.951 | 0.946 | 0.922 | 0.923 |
| 3256JPT+1KGP-JPT | 3,360 | 100 | 290.050 | 0.968 | 0.964 | 0.952 | 0.947 | 0.922 | 0.924 |
| 3256JPT+1KGP-EAS | 3,760 | 100 | 295.495 | 0.969 | 0.964 | 0.952 | 0.946 | 0.925 | 0.922 |
| 3256JPT+1KGP-EAS+EUR | 4,263 | 88.2 | 353.461 | 0.968 | 0.961 | 0.952 | 0.939 | 0.923 | 0.906 |
| 3256JPT+1KGP-EAS+EUR+AFR | 4,924 | 76.36 | 376.367 | 0.968 | 0.961 | 0.951 | 0.932 | 0.924 | 0.898 |
| 3256JPT+1KGP-EAS+EUR+AFR+AMR | 5,217 | 72.07 | 422.724 | 0.968 | 0.959 | 0.950 | 0.928 | 0.923 | 0.890 |
| 3256JPT+1KGP-All | 5,760 | 65.28 | 405.770 | 0.968 | 0.959 | 0.950 | 0.927 | 0.922 | 0.888 |

The 3,256 WGS samples of BBJ are included in all panels. 1KGP- followed by the ancestry represents the 1KGP subset. Minor allele frequency (MAF) is determined by the genotyping array.

**Supplementary Table 20. Number of variants using the simulated JPT-1KGP reference panels with different sizes and ancestral diversities, and WGS_993_ as the target sample (chr20).**

| Reference panel | Number of variants in reference panel | | Matches with HDS > 0.9 | | | | Variants with Rsq > 0.7 | | | |
| --- | --- | --- | --- | --- | --- | --- | --- | --- | --- | --- |
|  | All | Non-EAS | All | JPT_3256_-only | 1KGP-EAS-only | Non-EAS | All | JPT_3256_-only | 1KGP-EAS-only | Non-EAS |
| 100JPT | 266,077 | * | * | * | * | * | 135,205 | * | * | * |
| 500JPT | 476,580 | * | * | * | * | * | 177,631 | * | * | * |
| 1000JPT | 626,682 | * | * | * | * | * | 210,549 | * | * | * |
| 1500JPT | 732,311 | * | * | * | * | * | 232,098 | * | * | * |
| 2000JPT | 813,471 | * | * | * | * | * | 248,100 | * | * | * |
| 2500JPT | 881,067 | * | * | * | * | * | 260,145 | * | * | * |
| 3256JPT | 965,825 | * | 95,472,821 | 26,607 | * | * | 272,567 | 11,846 | * | * |
| 3256JPT+1KGP-JPT | 967,022 | * | 95,493,490 | 26,376 | * | * | 273,694 | 11,920 | * | * |
| 3256JPT+1KGP-EAS | 992,986 | * | 95,433,285 | 26,178 | 164 | * | 272,283 | 12,117 | 195 | * |
| 3256JPT+1KGP-EAS+EUR | 1,109,532 | 116,546 | 94,988,860 | 25,303 | 156 | 529 | 266,228 | 11,806 | 178 | 517 |
| 3256JPT+1KGP-EAS+EUR+AFR | 1,413,327 | 420,341 | 95,032,512 | 25,650 | 157 | 536 | 267,369 | 11,967 | 179 | 586 |
| 3256JPT+1KGP-EAS+EUR+AFR+AMR | 1,428,226 | 435,240 | 94,744,347 | 24,964 | 151 | 561 | 262,468 | 11,706 | 172 | 596 |
| 3256JPT+1KGP-All | 1,472,263 | 479,277 | 94,718,393 | 25,473 | 150 | 586 | 265,170 | 12,053 | 175 | 620 |

The number of JPT3256-only and 1KGP-EAS-only variants was 73,962 and 7,656. (*) denotes not available. 100–3256 JPT represents the number of JPT samples in the reference panel; 1KGP- followed by the ancestry represents the 1KGP subset.

**Supplementary References**

1. Nagai A, Hirata M, Kamatani Y, et al. Overview of the BioBank Japan Project: Study design and profile. J Epidemiol 2017;27(3S):S2–S8

2. Hirata M, Kamatani Y, Nagai A, et al. Cross-sectional analysis of BioBank Japan clinical data: A large cohort of 200,000 patients with 47 common diseases. J Epidemiol 2017;27(3S):S9–S21

3. Akiyama M, Ishigaki K, Sakaue S, et al. Characterizing rare and low-frequency height-associated variants in the Japanese population. Nat Commun 2019;10:4393

4. Flanagan J. Impact of reference panel choice on imputation into genome-wide association studies of complex human traits [PhD dissertation]. Liverpool: University of Liverpool, 2022. https://livrepository.liverpool.ac.uk/3155731.

5. Das S, Forer L, Schönherr S, et al. Next-generation genotype imputation service and methods. Nat Genet 2016;48:1284–1287

6. Das S, Abecasis GR, Browning BL. Genotype Imputation from Large Reference Panels. Annu Rev Genomics Hum Genet 2018;19:73–96

7. Li Y, Willer CJ, Ding J, et al. MaCH: using sequence and genotype data to estimate haplotypes and unobserved genotypes. Genet Epidemiol 2010;34:816–834

8. Das S. Next Generation of Genotype Imputation Methods [PhD dissertation]. Ann Arbor (MI): University of Michigan, 2017. https://deepblue.lib.umich.edu/handle/2027.42/138466.

9. Marchini J, Howie B. Genotype imputation for genome-wide association studies. Nat Rev Genet 2010;11:499–511

10. Zheng HF, Rong JJ, Liu M, et al. Performance of genotype imputation for low frequency and rare variants from the 1000 genomes. PLoS One 2015;10:e0116487

11. Hanks SC, Forer L, Schönherr S, et al. Extent to which array genotyping and imputation with large reference panels approximate deep whole-genome sequencing. Am J Hum Genet 2022;109:1653–1666

12. Oscanoa J, Sivapalan L, Gadaleta E, et al. SNPnexus: A web server for functional annotation of human genome sequence variation (2020 update). Nucleic Acids Res 2020;48:W185–W192

13. Jin Y, Terhorst J. The solution path of the Li-Stephens haplotype copying model. bioRxiv 2022;502674

14. Surakka I, Sarin A-P, Ruotsalainen SE, et al. The rate of false polymorphisms introduced when imputing genotypes from global imputation panels. bioRxiv 2016;080770
